# In Vitro Evaluation of Colistin Conjugated with Chitosan-Capped Gold Nanoparticles as a Possible Formulation Applied in a Metered-Dose Inhaler

**DOI:** 10.3390/antibiotics13070630

**Published:** 2024-07-06

**Authors:** Narumon Changsan, Apichart Atipairin, Poowadon Muenraya, Rutthapol Sritharadol, Teerapol Srichana, Neelam Balekar, Somchai Sawatdee

**Affiliations:** 1 College of Pharmacy, Rangsit University, Pathum Thani 12000, Thailand; narumon.c@rsu.ac.th; 2 School of Pharmacy, Walailak University, Thasala 80160, Nakhon Si Thammarat, Thailand; apichart.at@mail.wu.ac.th (A.A.); poowadon.me@wu.ac.th (P.M.); 3 Drug and Cosmetics Excellence Center, Walailak University, Thasala 80160, Nakhon Si Thammarat, Thailand; 4 Department of Pharmaceutics and Industrial Pharmacy, Faculty of Pharmaceutical Sciences, Chulalongkorn University, Bangkok 10330, Thailand; rutthapol.s@chula.ac.th; 5 Drug Delivery System Excellence Center, Department of Pharmaceutical Technology, Faculty of Pharmaceutical Sciences, Prince of Songkla University, Hat Yai 90112, Songkhla, Thailand; teerapol.s@psu.ac.th; 6 College of Pharmacy, IPS Academy, Indore 452012, Madhya Pradesh, India; neelambalekar@gmail.com

**Keywords:** antibacterial activity, colistin sulfate, controlled release, gold nanoparticle, pulmonary drug-delivery system, release kinetic model

## Abstract

Inhaled colistin is used to treat pneumonia and respiratory infections through nebulization or dry powder inhalers. Nevertheless, the development of a metered-dose inhaler (MDI) for colistin, which could enhance patient convenience and treatment efficacy, has not yet been developed. Colistin is known for its ability to induce cellular toxicity. Gold nanoparticles (AuNPs) can potentially mitigate colistin toxicity. Therefore, this study aimed to evaluate the antimicrobial effectiveness of colistin conjugated with chitosan-capped gold nanoparticles (Col-CS-AuNPs) and their potential formulation for use with MDIs to deliver the aerosol directly to the deep lung. Fourier-transform infrared spectroscopy, nuclear magnetic resonance, and elemental analysis were used to characterize the synthesized Col-CS-AuNPs. Drug release profiles fitted with the most suitable release kinetic model were evaluated. An MDI formulation containing 100 µg of colistin per puff was prepared. The aerosol properties used to determine the MDI performance included the fine particle fraction, mass median aerodynamic diameter, and geometric standard deviation, which were evaluated using the Andersen Cascade Impactor. The delivered dose uniformity was also determined. The antimicrobial efficacy of the Col-CS-AuNP formulation in the MDI was assessed. The chitosan-capped gold nanoparticles (CS-AuNPs) and Col-CS-AuNPs had particle sizes of 44.34 ± 1.02 and 174.50 ± 4.46 nm, respectively. CS-AuNPs effectively entrapped 76.4% of colistin. Col-CS-AuNPs exhibited an initial burst release of up to 60% colistin within the first 6 h. The release mechanism was accurately described by the Korsmeyer–Peppas model, with an R^2^ > 0.95. The aerosol properties of the Col-CS-AuNP formulation in the MDI revealed a high fine particle fraction of 61.08%, mass median aerodynamic diameter of 2.34 µm, and geometric standard deviation of 0.21, with a delivered dose uniformity within 75–125% of the labeled claim. The Col-CS-AuNP MDI formulation completely killed *Escherichia coli* at 5× and 10× minimum inhibitory concentrations after 6 and 12 h of incubation, respectively. The toxicity of CS-AuNP and Col-CS-AuNP MDI formulations in upper and lower respiratory tract cell lines was lower than that of free colistin. The stability of the Col-CS-AuNP MDI formulation was maintained for at least 3 months. The Col-CS-AuNP MDI formulation effectively eradicated bacteria over a 12-h period, showing promise for advancing lung infection treatments.

## 1. Introduction

Colistin, an effective antibacterial agent, demonstrates excellent bactericidal activity against aerobic Gram-negative pathogens, including *Acinetobacter baumannii*, *Klebsiella pneumoniae*, *Pseudomonas aeruginosa*, *Escherichia coli*, and other *Enterobacteriaceae* commonly associated with life-threatening infections [1]. However, colistin shows no activity against Gram-positive bacteria and most anaerobes [2]. Initially used in the 1950s, it was later withdrawn from the market because of its potential side effects, such as nephrotoxicity and neurotoxicity [3]. Colistin interacts with the lipid A component of lipopolysaccharide (LPS) in the outer membrane of Gram-negative bacteria. This interaction disrupts the calcium and magnesium bridges, stabilizing LPS and leading to detergent-like permeabilization of the bacterial outer membrane. Colistin, inserted through these fissures into the outer bacterial membrane, causes self-promoted uptake, compromising the integrity of the inner membrane and resulting in bacterial cell death [2].

Multidrug-resistant (MDR) Gram-negative bacteria that cause respiratory tract infections have been associated with increased mortality and morbidity rates [4]. Intravenous colistin administration is commonly used as rescue therapy for MDR Gram-negative lung infections. However, its polycationic/hydrophilic structure limits its penetration into the lungs, posing challenges in treating infections caused by Gram-negative pathogens exhibiting high minimum inhibitory concentrations (MICs). In addition, intravenous administration of colistin showed a high incidence of nephrotoxicity and neurotoxicity [5]. Direct delivery of colistin through the pulmonary route may be a promising alternative to intravenous administration, as it minimizes systemic exposure while delivering the drug precisely to the target site. In principle, this increases antimicrobial efficacy, decreases antimicrobial resistance, and provides a dose-sparing strategy for reducing colistin-induced nephrotoxicity and neurotoxicity [6,7]. The effectiveness of inhaled colistin, administered by nebulization or a dry powder inhaler, has been assessed for the treatment of pneumonia and respiratory infections. These results demonstrate that colistin administration via inhalation could effectively deliver colistin to treat MDR respiratory tract infections [8,9,10,11,12]. However, a metered-dose inhaler (MDI) for colistin has not yet been developed. The advantages of MDI devices include portability and low resistance, which are suitable for treating children and patients with decreased lung function [13].

Gold nanoparticles (AuNPs), sized 1–100 nm, can be chemically or naturally produced [14]. AuNPs are currently used as drug-delivery systems in numerous biomedical applications, particularly in the pharmaceutical industry. Their popularity stems from their ease of synthesis, stability, and non-toxic nature [15]. Additionally, they exhibit diverse antimicrobial properties and can enhance the antimicrobial activity of drugs due to gold ions [16,17,18]. However, AuNPs act as antibiotic carriers to enhance their bactericidal effects [18]. Conjugating metallic nanoparticles with antibiotics enhances the antibacterial properties of the drug while reducing side effects and lowering dose requirements [19,20]. This approach also minimizes the risk of developing bacterial resistance [21]. Given the dose-dependent side effects of colistin, using a lower dose to achieve the same therapeutic results would benefit patients. In a study conducted by Fuller et al. (2020), the use of AuNPs as colistin carriers was investigated [22]. Conjugation of colistin to anionic AuNPs exhibited a heightened antibacterial effect compared to colistin alone, leading to a six-fold reduction in the MIC against *E. coli* [22]. Therefore, assuming that colistin can bind to AuNPs, these particles are expected to exert antibacterial effects similar to those of the drug when used alone, albeit at lower doses [22]. AuNPs enhance the bactericidal activity of colistin and also increase the contact surface area of the drug with bacteria, thereby improving the ability of colistin to attach to the cell wall and eliminate bacteria [21]. Additionally, conjugation with AuNPs reportedly reduces the cell toxicity caused by colistin while maintaining the therapeutic effects [23]. AuNP-conjugated drugs have the potential for sustained targeted drug delivery and long-term release of hydrophobic compounds [24,25,26,27,28,29,30,31,32]. Therefore, the development of AuNPs as a pulmonary drug-delivery system holds considerable promise.

Chitosan is a promising biopolymer frequently used for its superior biocompatibility, biodegradability, antibacterial activity, non-toxicity, and financial benefits [33,34]. In an acidic aqueous solution, the protonated chitosan backbone (with positively charged NH_3_^+^ on the chitosan polymer) caused mutual repulsion swelling and enhanced the solubility of chitosan [34,35]. Owing to the strong affinity of gold for amino (—NH_2_), cyano (—CN), and thiol (—SH) functional groups, the presence of the —NH_2_ group in chitosan molecules increases their affinity for AuNPs [36]. Furthermore, chitosan was observed to be a good self-assembling polymer that acts as an effective reducing/stabilizing agent without requiring additional externally added reducing agents. The mechanism via an electrostatic force involves the bonding of positively charged —NH_3_^+^ with oppositely charged AuCl_4_. Moreover, when combined with metallic nanoparticles, chitosan exhibits synergistic antibacterial activity [37].

The advancement in nanotechnology-assisted MDIs has broadened their utility, offering benefits such as targeted drug delivery, rapid onset of action, achieving high drug concentrations, reduced dosage, lower systemic toxicity, and avoidance of first-pass metabolism [38]. Despite these advantages, incorporating AuNPs into MDI development has not been extensively explored, possibly because of concerns regarding the safety, toxicity, and inflammation associated with AuNP exposure [39,40]. Despite Au being biocompatible, it degrades slowly within the body [41]. According to a recent study, AuNPs were distributed bilaterally and in a dose-dependent manner in animals without causing short-term pain or airway irritation. AuNPs are specifically administered to human lung fibroblasts instead of abdominal organs, providing a non-invasive and targeted therapeutic alternative for long-term respiratory diseases [42]. However, toxicological data on the exposure of whole organisms to AuNPs via inhalation remain insufficient and controversial. The challenge in drawing conclusions regarding the safety of these AuNPs arises from the disparate data available stemming from variations in exposure conditions, sample preparations, cell lines used, and assays employed across different studies [40,43].

Conjugating colistin with AuNPs and developing it as an MDI formulation may enhance its antimicrobial activity, improve the colistin cytotoxicity profile, and promote a longer duration of action. In this study, the antimicrobial activity and cytotoxicity of colistin in AuNPs and its potential to be used with MDI were assessed. The goal of using AuNPs containing antibiotics in the MDI dosage form, such as Col-CS-AuNPs, is to enhance the effectiveness of existing antibiotics while also maintaining the high drug concentration and activity for a longer period to kill bacteria in the lungs while oral or injectable dosage forms are difficult to access to lower lung infection.

## 2. Results and Discussion

### 2.1. Synthesis of Colistin Conjugated with Chitosan-Capped Gold Nanoparticles (Col-CS-AuNPs)

The HAuCl_4_ solution displayed a strong yellow color (Figure S1A). Gold was reduced by chitosan, causing the color of the solution to shift from its original color to a clear red wine color when shrunk to the nanoscale (Figure S1B), suggesting the formation of chitosan-capped gold nanoparticles, as reported by Mohan et al. (2018) [44].

Subsequent conjugation of colistin with CS-AuNPs, facilitated by glutaraldehyde as the linker of the conjugation system, yielded a reddish-purple solution, which was slightly brighter than the CS-AuNPs (Figure S1C). The color of AuNPs changed depending on the particle shape and size due to the phenomenon of localized surface plasmon resonance (LSPR) [45,46]. As the size of the gold nanosphere increased, a considerable shift to the far-red region of the spectrum was observed for AuNPs [46]. The HAuCl_4_ solution displayed no Tyndall effect, indicating no light scattering by the particles in the colloidal system, thereby confirming a true solution. Conversely, both CS-AuNPs and Col-CS-AuNPs exhibited the Tyndall effect, confirming their status as colloidal solutions.

UV-Vis spectra of HAuCl_4_ showed an absorbance peak of 331 ± 0.3 nm, and CS-AuNPs showed a lower absorbance peak at 300 ± 0.2 and 515 ± 0.3 nm. Col-CS-AuNPs showed absorbance peaks at 300 ± 0.6 nm and 528 nm. This shift in absorbance from 515 to 528 nm indicated a particle size larger than that of the CS-AuNPs (Figure S2).

### 2.2. Physicochemical Properties of Col-CS-AuNPs

The hydrodynamic particle sizes and zeta potentials of the AuNPs measured using dynamic light scattering (DLS) are listed in Table 1. The size of CS-AuNPs was approximately 44.34 ± 1.02 nm. When colistin molecules were bound to CS-AuNPs, known as Col-CS-AuNPs, the hydrodynamic particle size increased to 174.50 ± 4.46 nm. This increased size, which was slightly larger than the typical size range of metallic nanoparticles (10–100 nm) [47], can be attributed to the role of glutaraldehyde as a linker, aiding in combining chitosan-capped gold nanoparticles with colistin molecules. Although the particle size of Col-CS-AuNPs was slightly larger, the size distribution remained normal and narrow, similar to that of CS-AuNPs before conjugation with colistin (Figure S3). The polydispersity index (PDI) indicates the quality of the size distribution. An index value smaller than 0.05 indicates high monodispersity, whereas a PDI value larger than 0.7 indicates a broad particle size distribution [48]. The PDI values of CS-AuNPs and Col-CS-AuNPs were 0.22 and 0.10, respectively, indicating that both CS-AuNPs and Col-CS-AuNPs were monodispersed particles.

**Figure 1:**
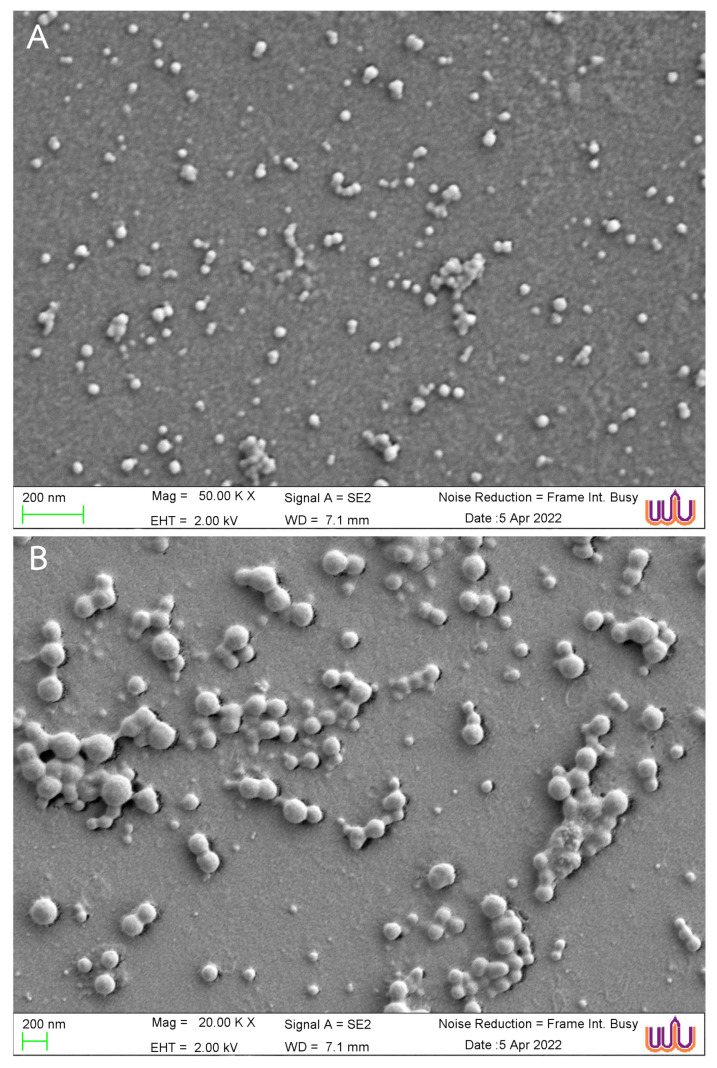
The SEM images of CS-AuNPs (**A**) and Col-CS-AuNPs (**B**).

Scanning electron microscopy (SEM) images of the morphology of CS-AuNPs and Col-CS-AuNPs with smooth surfaces are shown in Figure 1. The CS-AuNPs exhibited a spherical shape with a particle size of 27.3 ± 4.8 nm as measured using ImageJ software; however, the Col-CS-AuNPs had a larger particle size of 125.0 ± 22.1 nm.

These results indicated that the particle size analysis conducted using DLS, specifically the hydrodynamic diameter of both CS-AuNPs and Col-CS-AuNPs, did not accurately represent the effective size of the nanoparticle core. The hydrodynamic diameter obtained from the DLS of CS-AuNPs and Col-CS-AuNPs was larger than the size observed using SEM because DLS analysis typically measures both the particle core and shell. DLS typically measures the particle size in a water medium, where ligands, ions, and solvated water molecules form the particle shell, known as the electrical double layer. In contrast, SEM images particle morphology by detecting reflected electrons from the dry surface of the particle, where the shell has collapsed due to the drying process [49,50].

The zeta potential value of CS-AuNPs was +1.13 ± 0.01 mV, and the zeta potential of Col-CS-AuNPs was increased to +4.97 ± 1.05 mV. The conjugation of colistin to CS-AuNPs resulted in an increased zeta potential, likely attributable to the positive charge of colistin and the subsequent increase in the cationic charge, particularly abundant in the —NH_2_ group of the molecules.

The effects of chitosan concentration and molecular weight of the synthesized CS-AuNPs were determined according to Fuster et al. (2020) using a medium molecular weight of chitosan with a concentration of 0.1% *w*/*v*. The particle size of the CS-AuNPs was comparable to that reported in a previous study, except for the zeta potential value, which was one-tenth of that previously reported [51,52].

**Figure 2:**
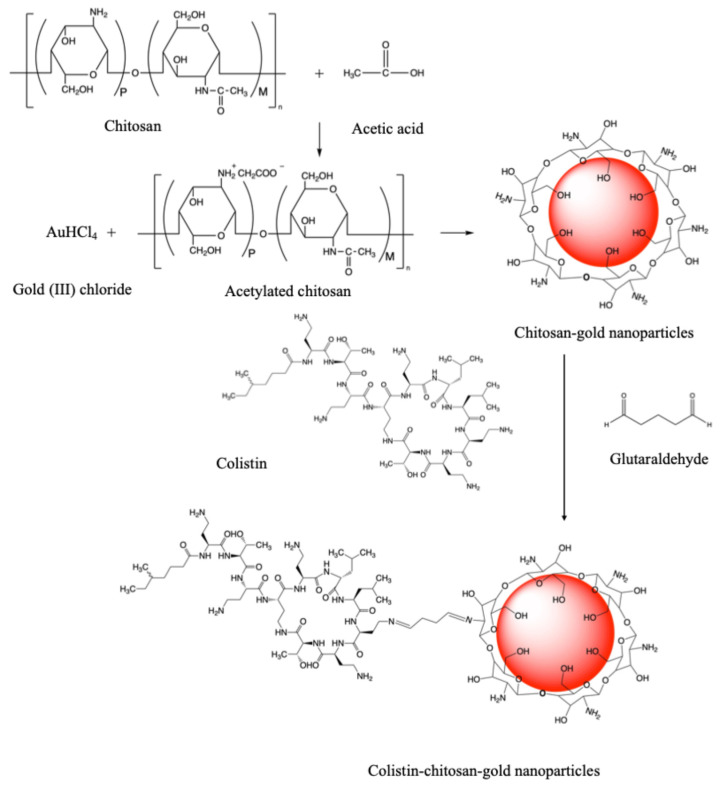
Schematic diagram of the synthesis of colistin conjugated with chitosan-capped gold nanoparticles (Col-CS-AuNPs).

The FT-IR spectra of colistin, CS-AuNPs, and Col-CS-AuNPs are shown in Figure S4. Colistin shows a characteristic band of the primary and secondary amide (N-H) at approximately 3300 (stretching) and 1536 cm^−1^ (bending). Additionally, the N-H bending occurred at 1640–1550 cm^−1^ for both primary and secondary amides. C=O stretching occurred at 1680–1630 cm^−1^. The free O-H stretch had a sharp peak at 3650–3600 cm^−1^. The hydrogen-bonded O-H band showed a broad peak at 3400–3300 cm^−1^ of the O-H stretch. This peak overlapped with that of the primary amide [53,54]. The FT-IR peaks of the CS-AuNPs and Col-CS-AuNPs did not show the characteristic peaks of C=O and —NH_2_, indicating a possible coupling reaction between colistin and chitosan. Glutaraldehyde acted as a bridge to link CS-AuNPs and colistin via an imine bond (N=CH) between its aldehyde groups and both amino groups, as shown by the transmission peak at 1637–1639 cm^−1^ (please refer to the schematic diagram in Figure 2). Stretching in an imine leads to variable-intensity absorption in the range of 1690–1640 cm^−1^ [54]. The characteristic —C=N peak differed from the C=O and —NH peaks of colistin, preliminarily indicating the formation of Col-CS-AuNPs. Additionally, a weak peak at 2255 cm^−1^ revealed a nitrile IR characteristic peak, which may have occurred during the synthesis process.

To verify the identity of the synthesized Col-CS-AuNPs, nuclear magnetic resonance (NMR) was employed to determine the interaction between the aldehyde group (—HC=O) of glutaraldehyde, which served as a crosslinker and the amino group (—NH_2_) of colistin and chitosan-capped AuNPs. The ^1^H NMR spectra of colistin and Col-CS-AuNPs are presented in Figure S5, and the chemical shifts are summarized in Table S1. Notably, the shifts in the Col-CS-AuNPs spectrum, when compared to colistin, indicate remarkable interactions primarily with the amines (both primary and secondary) in colistin, which show marked spectral changes. Additionally, interactions appear to involve the amine functions of chitosan and alcohol functions.

CHN/O elemental analysis was used to confirm the elemental composition during the synthesis of Col-CS-AuNPs (Table S2). Colistin components were found to be 52.1% carbon (C), 8.1% hydrogen (H), 18.1% nitrogen (N), and 21.7% oxygen (O). For CS-AuNPs, the values were 44.1% C, 6.8% H, 8.1% N, and 41.0% O. Col-CS-AuNPs elemental composition revealed 45.9% C, 7.6% H, 10.2% N, and 36.3% O. The bond formation between colistin and CS-AuNPs during synthesis was responsible for these proportional differences in elements.

The colistin content in metallic nanoparticles was analyzed using high-performance liquid chromatography (HPLC), following the conditions reported in a previous study, which had been validated for high specificity for colistin [53]. This analytical method was used to determine colistin in the synthesized AuNPs. The analytical method for colistin was validated using eight parameters: specificity, range, linearity, inter- and intra-day precision, accuracy, limit of detection (LOD), limit of quantification (LOQ), and robustness. For the identification of colistin, the standard exhibited a retention time of 9.5 min, and the samples were eluted at the same retention time (Figure S6). The LOD and LOQ of colistin were 0.46 and 1.57 μg/mL, respectively. The percentage of colistin recovery was 98.97% ± 0.12%, with a relative standard deviation of 0.25%, which was deemed acceptable for accuracy and precision. The HPLC system demonstrated linearity between 1 and 200 μg/mL. Therefore, in this experiment, all parameters used to validate the analytical method for assessing colistin were accepted in accordance with the International Council for Harmonization (ICH) recommendations [55].

A schematic diagram of the chemical reactions involving colistin, HAuCl_4_, and chitosan to produce Col-CS-AuNPs is shown in Figure 2. In an acidic aqueous solution, the protonated chitosan backbone (with positively charged NH_3_^+^ on the chitosan polymer) caused mutual repulsion swelling and enhanced the solubility of chitosan upon binding to the surface of AuNPs [34,35]. Chitosan was observed to be a good self-assembling polymer that acts as an effective reducing/stabilizing agent without requiring additional externally added reducing agents. This mechanism involves the bonding of positively charged —NH_3_^+^ with CS-AuCl_4_ via an electrostatic force with oppositely charged AuCl_4_. Chitosan was adsorbed on the ion surface during the initiation phase, and the reduction reaction resulted in binding. Novel Col-CS-AuNPs were synthesized using a modified chemical reduction method based on previous studies [22,36,41,52,56,57]. Glutaraldehyde was used as a linker for chitosan and colistin in this study, based on the —COH functional group, which is easily crosslinked, as previously described [58,59].

The entrapment efficiency of Col-CS-AuNPs was 76.08% ± 7.04%, showing high encapsulation efficiency. The percentage of entrapment efficiency is defined as the difference between the total drug load and the free or unencapsulated drug in the supernatant relative to the total drug load. Nevertheless, the abovementioned entrapment efficiency is a theoretical value that may not accurately reflect the actual value. This discrepancy can be attributed to various factors, including the extremely low quantity of free colistin, which cannot be exactly quantified. The formulation development of Col-CS-AuNPs in MDI was calculated using a drug content of 76.08% as the theoretical value (100% of the active ingredient).

### 2.3. Controlled Release of Col-CS-AuNPs and Kinetic Models Fitting

**Figure 3:**
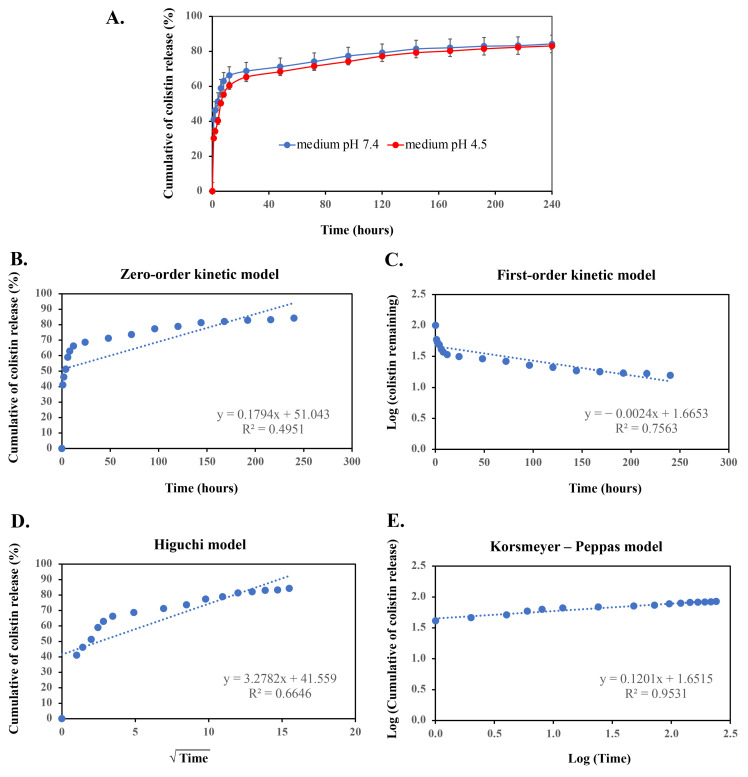
Cumulative colistin release (%) from Col-CS-AuNPs over ten days (**A**). Colistin release from Col-CS-AuNPs was fitted to kinetic models (**B**–**E**). Colistin release was fitted to each kinetic model: zero-order kinetic model by plotting cumulative % drug release vs. time (**B**), first-order kinetic model by plotting the logarithmic value of the remaining percent drug release vs. time (**C**), simplified Higuchi model by plotting cumulative % drug release vs. the square root of time (**D**), and Korsmeyer–Peppas model by plotting the log-cumulative % drug release vs. log time (**E**). The Korsmeyer–Peppas model showed a high correlation with R^2^ > 0.95. Dots represent the drug released at a specified time point, and the dashed line represents the regression line, as shown in each graph.

This experiment involved an in vitro release test to monitor the release time of the maximum dose of colistin by studying the 10-day release profile of the Col-CS-AuNPs. In a medium with a pH of 7.4—similar to the lung environment—over 50% of colistin was released from the Col-CS-AuNPs within the first 5 h, exhibiting a rapid release phase 5 h later (Figure 3A). Subsequently, the release rate stabilized at a nearly constant 65–85% over the remaining period. By the end of 10 days (240 h), colistin release had continued at 85%. However, inhaled particles that settle in the respiratory tract are generally eliminated within 3–24 h via intercellular or transcellular diffusion, mucociliary clearance, and pinocytotic processes [60,61]. The prolonged release of Col-CS-AuNPs may not be sustainable due to the respiratory tract clearance mechanism. Therefore, it is critical to maintain a sufficient concentration of colistin to preserve its antimicrobial activity [62]. For the Col-CS-AuNPs, the release of colistin reached 60% within 6 h, sufficiently exceeding the MIC of 2 μg/mL for *P. aeruginosa* [63], thus proving effective in treating bacterial infections in the lungs.

Furthermore, the environment of an inflammatory site typically exhibits low acidity when a bactericidal infection is present. Thus, we investigated the release of colistin, which was observed to be 2–7% lower at pH 4.5, relative to pH 7.4; however, the release profile remained consistent with that observed at pH 7.4. The colistin release profile of Col-CS-AuNPs showed a controlled release of colistin from the AuNPs. However, the synthesis method did not separate the bound and unbound colistin and CS-AuNPs. Therefore, this approach may result in the mixing of the two components of Col-CS-AuNPs. Thus, the immediate release in the first 12 h may be attributed to the chitosan coating or weak interactions among colistin, chitosan, and free colistin. In addition, the delayed release of a fraction of colistin could be related to the covalent bonding between colistin and the aldehyde functional group of glutaraldehyde. These bonds are strong and difficult to break, resulting in a slower release of colistin. Mathematical models can be used to investigate the kinetics and mechanisms involved in drug release from nanoparticles. The release curves illustrated in Figure 3B–E were fitted with four different models to determine which model had the highest correlation with the experimental findings (Table 2). The high correlation with the Korsmeyer–Peppas model (R^2^ = 0.9531) indicates that aggregate drug release from AuNPs can be modeled similarly to a polymeric system undergoing degradation [27,64].

In this experiment, we analyzed the 10-day drug release profile to determine the duration required for complete release of the drug. We found that colistin was not released completely. Figure 3A reveals that the Col-CS-AuNPs system released almost 80% of colistin at 12 h, potentially marking the peak of drug release.

The influence of ethanol and sorbitan monooleate used as excipients in MDI formulation may slightly impact drug release due to the small amount of ethanol and sorbitan monooleate. Colistin sulfate is freely soluble in water but slightly soluble in ethanol [65]. Therefore, Col-CS-AuNPs remained in suspension within the product concentrate. Sorbitan monooleate, acting as a solubilizing enhancer, was used at a low concentration (0.05%), which is unlikely to significantly affect drug release. A high concentration of ethanol reportedly induces the aggregation of AuNPs [66]. However, an ethanol content of 10% in the Col-CS-AuNP MDI formulation was considered to have negligible effects on drug aggregation, as evidenced by the content uniformity parameter. In addition, sorbitan monooleate added to the formula also helped prevent agglomeration [67].

### 2.4. Development of the Col-CS-AuNP MDI Formulations

The dose of the Col-CS-AuNP MDI formulations was determined by integrating the aerosol properties of the MDI on pulmonary deposition with the MIC values. To combat bacterial lung infections effectively, colistin concentrations in lung fluid must exceed an MIC of 2 μg/mL. The extravascular lung fluid volume was estimated to be 10 mL based on approximately deep lung fluid [68,69]. Multiplying this volume by the desired colistin concentration (MIC) of 2 μg/mL yields the required colistin dose of 20 µg. However, when administering the MDIs, approximately 20–25% of the emitted dose from the canister reaches the target site [67]. Consequently, a dose of 100 µg/dose was employed in this study, which accounted for a minimum of 20% of deposition in the lung; the colistin concentration in the lung fluid should be higher than the colistin MIC value of 2 μg/mL.

The therapeutic efficacy of the inhalation persisted despite potential variations in the estimated dose. Accurate dosing requires a comprehensive understanding of the pharmacokinetics of the inhalation formulations. However, the estimated dose of our method differs from other inhaled administration methods of colistin, such as nebulization and DPIs. Typically, the recommended dose of colistin is 150 mg via inhalation, following an intravenous colistin loading dose, which has shown improved microbiological outcomes without nephrotoxicity [3]. Several studies have reported average doses of aerosolized colistin of approximately 80 mg/70 kg/day, yielding clinical responses to pulmonary infections comparable to those of intravenous administration [2].

For our calculated dose, we anticipate that the Col-CS-AuNP MDI can effectively deliver colistin deep into the lung, ensuring its concentration remains above the MIC necessary to eliminate bacteria. If the initial dose of 100 µg per puff is insufficient, additional inhalations may be required to reach the MIC. Further studies on animal and human subjects are necessary to confirm that lung drug concentrations exceed the MIC.

The freeze-dried Col-CS-AuNPs contained 2.84% colistin by weight, according to the colistin assay. To achieve approximately 20 mg of colistin per canister, with 200 puffs delivering 100 µg of colistin per puff, 704 mg of freeze-dried Col-CS-AuNPs were required. For practicality, the weight of the freeze-dried Col-CS-AuNPs used in the MDI formulation was set to 700 mg (0.7 g).

The colistin content assay conducted on the Col-CS-AuNP MDIs revealed a mean colistin content of 98.64% ± 2.34% of the indicated amount, approaching 100%. The uniformity of the delivered dose was 95.05% ± 4.08%, falling within the acceptable range of 75–125% for the beginning and end of unit life (beginning of unit life [BOU] and end of unit life [EOU]), as per pharmacopeia standards.

The inclusion of ethanol in the MDI formulation can affect the delivery characteristics of MDIs [67,70]. Sorbitan monooleate serves multiple purposes in the formulation, including enhancing drug solubility, preventing valve sticking, irreversible particle agglomeration, drug particles adhering to the container walls and valve components, and reducing the separation rate between the drug and propellant system [70]. Ethanol is used as a vehicle in the concentration process of the MDI formulation. The solvent evaporates rapidly when the drug is released from the MDI device. Hence, the presence of antibacterial effects and respiratory toxicity related to ethanol are expected to be minimal. Nonetheless, caution is advised regarding the high ethanol content in AuNPs, as it may affect the aggregation of nanoparticles [66].

### 2.5. Aerosol Properties of Col-CS-AuNP MDI Formulations

**Figure 4:**
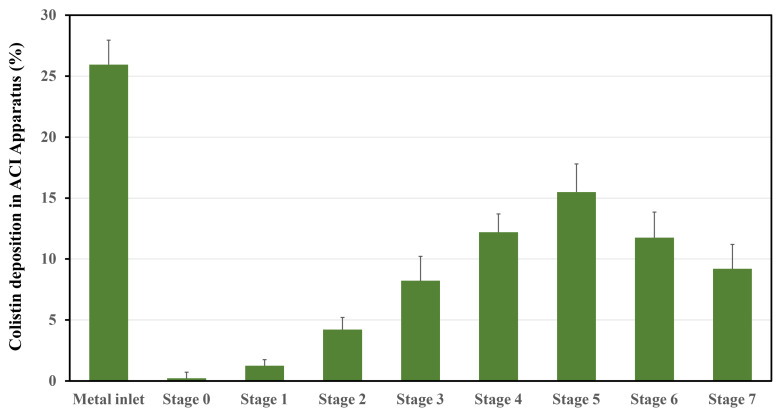
Colistin deposition (%) in each stage of the Andersen Cascade Impactor (ACI, USP apparatus 1) determined at an airflow rate of 28.3 L/min. Results are presented as mean ± SD, *n* = 6. Cut-off diameters are as follows: Stage 0 (9.0 μm), Stage 1 (5.8 μm), Stage 2 (4.7 μm), Stage 3 (3.3 μm), Stage 4 (2.1 μm), Stage 5 (1.1 μm), Stage 6 (0.7 μm), and Stage 7 (0.4 μm).

The aerosol properties of the Col-CS-AuNP MDI are detailed in Table 3, and the aerodynamic particle size distribution is shown in Figure 4. Approximately 25% of the administered Col-CS-AuNPs were deposited in the oral cavity, specifically in the metal inlet, which was the highest deposition observed across all stages. This suggests a notable loss of Col-CS-AuNPs in the oral cavity. Furthermore, Col-CS-AuNPs with a diameter > 5 μm were detected in small amounts in stages 0–1. However, a greater deposition of Col-CS-AuNPs was observed in stages 2–7 (ranging from >5% to 15% for each stage; Figure 4), which corresponds to a target site, the lower respiratory tract, and indicates that Col-CS-AuNPs are capable of reaching the small airways.

Fine particles, within the size range of 0.4–4.7 μm and deposited up to stage 2 of the Andersen Cascade Impactor (ACI), demonstrate potential for delivery to the lower lung region [71,72,73,74]. The fine particle fraction (FPF) of the Col-CS-AuNP MDI was 61.08% ± 2.03%, and the fine particle dose (FPD) was 61.08 ± 2.03 µg of colistin. The mass median aerodynamic diameter (MMAD) was 2.34 ± 1.01 µm, indicating its suitability for lower airway delivery. The geometric standard deviation (GSD) was 0.21 ± 0.02, which was less than 1.2, confirming that aerosol particles were monodispersed [75]. However, based on the release profile of Col-CS-AuNPs, 60% of colistin was released within 12 h before clearance by airway mechanisms. This indicated that approximately 36 µg of colistin (100 µg theoretical dose × 60% drug released × 60% FPD) was delivered and released to the airway surfaces. However, the colistin concentration should remain above the MIC of 2 μg/mL, which is effective against bacterial infections in the lungs. If necessary, the Col-CS-AuNP MDI (delivering 100 μg of colistin per puff) can be administered multiple times to achieve sufficient drug delivery. At this stage, the potential lower blood concentrations resulting from the controlled-release mechanism of colistin have not been considered. All delivered doses of colistin were within the range of 75–125% of the BOU and EOU according to the USP pharmacopeia acceptance criteria [76]. This demonstrated the consistency of the drug delivered from the device throughout its lifetime.

### 2.6. Antimicrobial Activity of Col-CS-AuNP MDI Product Concentrate

The MIC represents the lowest concentration of Col-CS-AuNPs required to prevent microbial growth. Owing to the challenges in MDI administration, the product concentrate was used instead of the MDI formulation, with the product concentrate being the formulation devoid of a propellant.

The results revealed that the MIC of colistin, Col-CS-AuNP used as an active pharmaceutical ingredient (API) in the formulation of the MDI, and Col-CS-AuNP formulation without propellant (calculated equivalent doses of colistin) were 4, 8, and 8 µg/mL, respectively (Table 4). Despite ethanol and sorbitan monooleate presented in the Col-CS-AuNP MDI product concentrate, Col-CS-AuNPs (API) and the product concentrate demonstrated the same antimicrobial activity with the same MIC. This indicated that the additives in the product concentrate did not have an effect on antimicrobial activity.

The Col-CS-AuNPs showed a two-fold higher MIC value than colistin alone. Due to the exposure of bacterial cells to the entire pure colistin, colistin exhibited greater potency than the Col-CS-AuNP formulation without propellant, as evidenced by the lower MIC obtained. Conversely, at 24 h of MIC observation, the drug release profile indicated that approximately 70% of colistin was released from the Col-CS-AuNP product concentrate, resulting in lower colistin presentation and subsequently reduced antimicrobial activity. Of note, different capping agents might also affect antibacterial action by altering drug release [77]. The study of Fuller et al. (2020) found that colistin conjugated with citrate-capped AuNPs had a lower MIC against *E. coli* than free colistin, while colistin conjugated with polydiallyldimethylammonium chloride-capped AuNPs exhibited no difference in MIC [22]. In our study, chitosan was used as a capping agent, and it was reported to increase bacterial growth at low concentrations, leading to a higher MIC than colistin alone [78]. CS-AuNPs in the form of a product concentrate, representing the blank particle, exhibited antibacterial activity at a high concentration, with an MIC of 128 µg/mL. This result coincides with previous findings on chitosan-based AuNPs, which also exhibited antibacterial and antifungal properties [79]. Chitosan serves as a stabilizer for AuNPs and also enhances the antibacterial activity when used to coat AuNPs [79]. The presumed inhibitory mechanism of chitosan involves the interaction between the positively charged chitosan and the negatively charged biomolecular residues on the surface of bacterial cells, which is particularly effective in acidic environments [79]. However, in this study, the positively charged chitosan in the Col-CS-AuNPs interacted with glutaraldehyde, consequently failing to enhance the antibacterial activity of the formulation.

**Figure 5:**
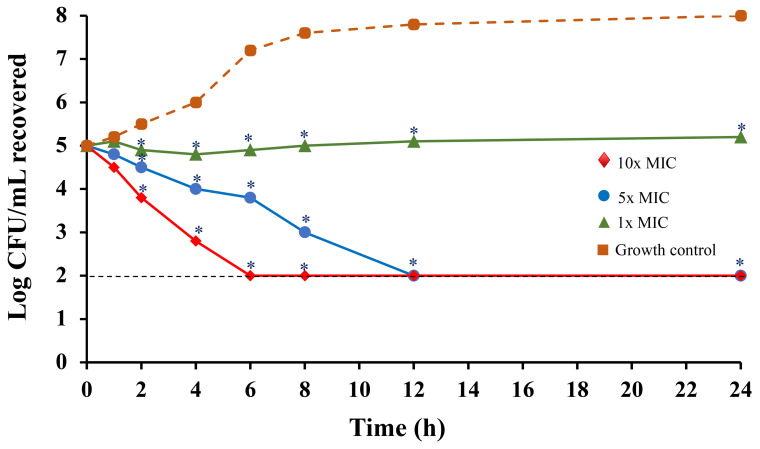
A time-kill kinetics curve of Col-CS-AuNP product concentrate of MDI formulation against *E. coli* at concentrations of 1×, 5×, and 10× MIC values. The samples were incubated with the tested bacteria at specified intervals (*n* = 3). The asterisk (*) indicates significant differences (*p*-value < 0.05) compared to treated and non-treated samples at the same intervals. The dashed line represents a limit of detection of 2 log CFU/mL.

The time-kill kinetics assay demonstrated that the Col-CS-AuNP formulation without propellant, at concentrations 10 and 5 times the MIC, considerably reduced the number of viable cells (colony-forming units [CFU]/mL) over time (Figure 5). Specifically, the number of viable *E. coli* cells (Gram-negative bacteria) decreased after 6 and 12 h. This reduction in bacterial viability was consistently significant compared to the antibacterial activity of the non-treated control group (*p* < 0.05) throughout the 24 h observation period. The reduction in the bacterial survival rate during 0–10 h at 10× and 5× MIC was related to the release profile of colistin, which was found to be rapidly released within the first 10 h. This rapid release might be due to the effect of weakly bound colistin from the Col-CS-AuNPs. Substantial cell reduction was also observed after 10 h as a result of the strongly bound colistin being released. Additionally, the MIC results demonstrated that the Col-CS-AuNP formulation without propellant exhibited greater antibacterial activity than CS-AuNPs due to the presence and release of colistin, which effectively killed bacteria.

### 2.7. Cytotoxicity of Col-CS-AuNP MDI Formulations

**Figure 6:**
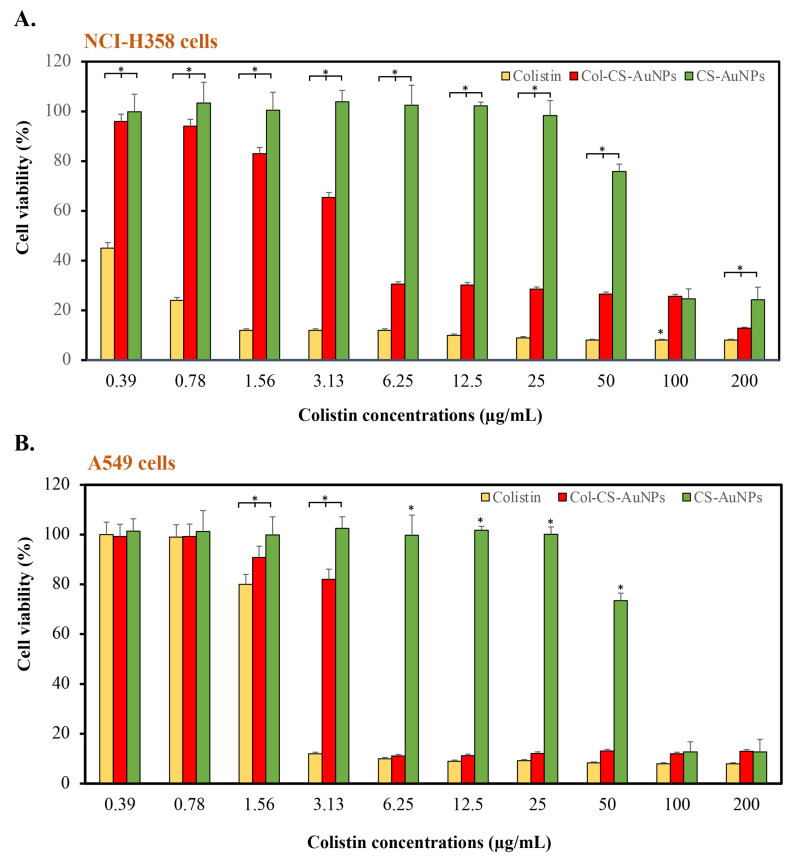
Percent viability of NCI-H358 cells (upper respiratory tract cell line) (**A**) and A549 cells (lower respiratory tract cell line) (**B**) to determine cytotoxic effect by MTT assay after incubation with colistin (orange bars), colistin conjugated with chitosan-capped gold nanoparticles metered-dose inhaler (Col-CS-AuNP MDI) formulation (red bars), and chitosan-capped gold nanoparticles (CS-AuNPs) used as blank (green bars). Data represent the mean ± standard deviation, *n* = 3. The asterisk (*) indicates significant differences (*p*-value < 0.05) between the groups.

The cytotoxicity of the Col-CS-AuNP MDI product concentrate (without propellant) intended for respiratory tract administration was tested using human lung adenocarcinoma (A549 cells) and Caucasian bronchioalveolar carcinoma cells (NCI-358), representing lower and upper respiratory tract epithelial cells, respectively. Figure 6A,B present the viability of NCI-358 and A549 cells, respectively, after exposure to colistin, Col-CS-AuNP MDI product concentrate, and blank CS-AuNP MDI product concentrate formulation. NCI-358 cells were more sensitive to colistin and Col-CS-AuNPs than A549 cells (type II alveolar epithelial cells), representing lower respiratory tract cells. Free colistin exhibited significant toxicity toward both examined cell types. Even at the lowest tested concentration of 0.39 μg/mL, the viability of NCI-H358 cells was less than 50%. However, A549 cells showed increased resistance to colistin, with a concentration of 3.13 μg/mL, resulting in cell viability below 80%.

The conjugation of colistin to CS-AuNPs reduced the toxicity of colistin against NCI-358 and A549 cells. The concentrations at which cell viability decreased below 80% were 3.13 and 6.25 μg/mL, respectively.

The blank-CS-AuNP MDI product concentrate exhibited the lowest toxicity, with 80% cell viability maintained upon exposure to concentrations of up to 50 μg/mL, calculated equivalent to the colistin-containing formulation. Meanwhile, at 2 μg/mL—the required MIC concentration on the lung surface—Col-CS-AuNPs caused the cell viability of NCI-358 and A549 to be higher than 60%.

The significant toxicity of Col-CS-AuNPs can be attributed to the release of free colistin from the formulation, as well as the positive charge impact of Col-CS-AuNPs. The increased positive charge of Col-CS-AuNPs resulted in greater interaction with negatively charged glycosaminoglycans on the surface of mammalian cells [40,80]. As a result, they could penetrate respiratory cells more efficiently than CS-AuNPs, which have a smaller positive charge than Col-CS-AuNPs. In our previous study, we performed a toxicity evaluation of colistin with AgNPs (Col-AgNPs) in human primary renal proximal tubule epithelial cell lines, indicating an average cell survival rate exceeding 80% at a Col-AgNP concentration of less than 32 μg/mL [53]. In the present study, Col-CS-AuNPs seemed to be more toxic than Col-AgNPs. However, the NCI-H358 and A549 cell lines are more sensitive than human epithelial cell lines [81].

Despite these findings, other clinical studies of aerosolized colistin have administered higher colistin doses than those in Col-CS-AuNP MDIs, with results showing effective treatment and good tolerance with few reported side effects [2,3,82]. Moreover, the recommended dose of 150 mg of colistin via inhalation, following an intravenous colistin loading dose, has been clinically applied and has demonstrated improved microbiological outcomes without nephrotoxicity or other side effects [3]. Nevertheless, to confirm the safety of the formulation, further in vivo studies on both animal and human subjects are warranted.

### 2.8. Stability of Col-CS-AuNP MDI Formulations

The stability assessment of the Col-CS-AuNP MDIs is summarized in Table 5. The colistin content in Col-CS-AuNPs exhibited a minor deviation of 2.79% ± 0.21% *w*/*w*, indicating <2% alteration from the original content after storage for 3 months at 2–8 °C. No significant alterations in appearance, particle size, or zeta potential were observed, confirming stability over the storage period. The stability of Col-CS-AuNP is corroborated by consistent zeta potential values and UV-visible red-shift observations [22]. We concluded that Col-CS-AuNPs should be stored at cold temperatures. However, the stability of the formulation under ambient and other temperature conditions was not investigated in this study, and further studies are required to determine the optimal storage conditions. The theoretical colistin content in Col-CS-AuNPs was 2.84% *w*/*w* when formulated as MDIs, with each puff delivering 100 µg. Aerosol properties following storage at 30 ± 2 °C and 75% ± 5% relative humidity remained suitable for respiratory application, though there was a slight increase in MMAD and a decrease in FPF compared to initial values. This deviation may be ascribed to Col-CS-AuNP aggregation during MDI preparation. These results confirmed the product’s stability for at least 3 months at room temperature in hot and humid climates [83]. While HFA propellants in MDIs can denature proteins [84,85,86], previous studies have shown protein stability in MDI formulations containing HFA [86,87]. However, the integrity and conformational stability of peptides and protein confirmations, which are crucial for their activity, should be further investigated.

## 3. Materials and Methods

### 3.1. Materials

Colistin sulfate, gold (III) chloride, and chitosan (medium molecular weight: 190,000–310,000 Da) were purchased from Sigma-Aldrich (St. Louis, MO, USA). Glutaraldehyde was purchased from ITW Reagents PanReac Applichem (Darmstadt, Germany). Sodium sulfate was obtained from Carlo Erba Reagenz (Emmendingen, Germany). All analytical-grade chemical reagents, including acetic acid, acetonitrile, methanol, and ethanol, were purchased from RCI Labscan Limited (Bangkok, Thailand). Additionally, ultra-pure hydrofluoroalkane 134a (HFA-134a) and 1,1,1,2-tetrafluoroethane (pharmaceutical grade) were purchased from Mexichem Fluor (Runcorn, UK). Further, *E. coli* TISTR 887 was purchased from the Thailand Institute of Scientific and Technological Research (TISTR; Khlong Luang, Pathum Thani, Thailand). Muller–Hinton (MH) agar and cation-adjusted Mueller–Hinton broth (CAMHB) were purchased from Gibco^TM^ (Thermo Fisher Scientific, Waltham, MA, USA). Finally, 3-(4,5-dimethylthiazol-2-yl)-2,5-diphenyl-tetrazolium bromide (MTT) was purchased from Sigma-Aldrich.

### 3.2. Synthesis of Col-CS-AuNPs

A modified version of a previously reported protocol was used to synthesize CS-AuNPs and Col-CS-AuNPs [51,56]. Briefly, a 10-mM gold (III) chloride (HAuCl_4_) solution was prepared by dissolving 170 mg of HAuCl_4_ in 50 mL of distilled water. Chitosan (0.5 g) was dissolved in a 1% acetic acid solution and adjusted to a volume of 50 mL, after which it was stirred at room temperature (25–30 °C) for 24 h. CS-AuNPs were prepared by combining 50 mL of 10 mM HAuCl_4_, 15 mL of chitosan solution, and 1 mL of 25% glutaraldehyde. The solution was heated at 70 °C for 15 min, resulting in a color change from yellow to wine-red. Thereafter, the solution was left to cool to room temperature. Col-CS-AuNPs were prepared in the same way as CS-AuNPs by adding colistin (10 mg/mL) together with 1 mL of 25% glutaraldehyde, serving as a conjugation system linker, into the mixture. The reaction mixture was vigorously stirred at 800 rpm for 60 min at room temperature (25–30 °C). The resulting CS-AuNPs or Col-CS-AuNPs were filtered using Whatman No. 1 filter paper, and the filtrate was refrigerated at 4 °C to stop the reaction. Thereafter, the filtrate was centrifuged at 10,000 rpm for 25 min at 4 °C, and the supernatants and precipitated samples were separately collected for further analysis. The obtained CS-AuNPs or Col-CS-AuNPs were dried to remove all solvents using a freeze-dryer (Martin Christ Gefriertrocknungsanlagen GmbH, Osterode am Harz, Germany). CS-AuNPs were used as a control.

### 3.3. Characterization of CS-AuNPs and Col-CS-AuNPs

#### 3.3.1. Appearance

The color and clarity of the CS-AuNP and Col-CS-AuNP solutions were visually examined, and the Tyndall effect was used to confirm the colloidal properties. An ultraviolet-visible (UV-Vis) spectrophotometer (Jasco Corporation, Tokyo, Japan) was used to record UV-Vis spectra in the 200–700-nm range.

#### 3.3.2. Particle Size Analysis and Zeta Potential

CS-AuNP and Col-CS-AuNP (0.1 g ± 5 mg) powders were weighed after freeze-drying using an analytical balance and resuspended in ultra-purified water to obtain a concentration of 200 μg/mL. Their zeta potential at 25 °C was determined via electrophoretic mobility, and their particle sizes and polydispersity index were measured at a 90° angle using a Zetasizer or DLS analyzer (Malvern Panalytical Ltd., Malvern, UK). For every sample, the experiment was performed in triplicate.

#### 3.3.3. Fourier-Transform Infrared Spectroscopy

Col-CS-AuNPs, CS-AuNPs (freeze-dried powder, 2 mg), and colistin powder were compressed using KBr at a ratio of 1:50 to produce a semi-transparent 2-mm disk. The attenuated total reflectance method in Fourier-transform infrared spectroscopy (FT-IR; Tensor 27; Bruker, Ettlingen, Germany) covering the wavelength range of 4000–400 cm^−1^ was used to identify the binding in Col-CS-AuNPs. CS-AuNPs were used as a control to compare the conjugated forms of colistin.

#### 3.3.4. Morphology Examined Using a Scanning Electron Microscope

A scanning electron microscope (Zeiss Merlin FEG-SEM; Carl Zeiss, Oberkochen, Germany) was used to determine the morphology of the CS-AuNPs and Col-CS-AuNPs. All samples were dried on glass over a carbon ribbon, which served as an electron conductor. A sputter coater was used to coat the sample surfaces with gold mist. An electron microscope with a magnification of 20,000× or 50,000× was employed. Particle size was determined using the ImageJ software program (US NIH, Bethesda, MD, USA) based on SEM images.

#### 3.3.5. Nuclear Magnetic Resonance Spectroscopy (NMR)

Col and Col-CS-AuNPs were dissolved in D_2_O. The 1H and 13C NMR spectra of both colistin and Col-CS-AuNPs were measured using an NMR spectrometer (Bruker Ascend500/Avance Neo, Bruker Corporation, MA, USA).

#### 3.3.6. Elemental Analysis

The C, H, N, and O content of colistin, CS-AuNPs, and Col-CS-AuNPs were analyzed using CHNS/O Analyzer 1 (Thermo Quest model FlashEA 2000, Thermo Fisher Scientific, Milan, Italy).

### 3.4. Analysis of Colistin Content in Col-CS-AuNPs

The colistin concentration in the Col-CS-AuNPs was determined using an HPLC system (Thermo Fisher Scientific). The analysis was performed following established procedures outlined in previous studies [53,88]. A reversed-phase stainless steel column (Trajan^®^ C18 HPLC column, 4.6 × 250 mm, 5 µm) was used as the stationary phase. The mobile phase consisted of a mixture of acetonitrile and 30 mM Na_2_SO_4_ pH 2.3 at a ratio of 24:76 by volume. The flow rate was 1 mL/min. A sample concentration of approximately 20 μg/mL and an injection volume of 50 µL were used. Colistin was detected at a wavelength of 215 nm to determine its content and eluted at a retention time of approximately 13 min. The analytical method was validated in accordance with the guidelines outlined by the Association of Southeast Asian Nations (ASEAN), considering parameters such as specificity, limit of detection, LOQ, linearity, range, precision, accuracy, and robustness [89]. A colistin standard solution was prepared for the standard curve by dissolving colistin in distilled water at concentrations ranging from 1 to 50 µg/mL. All samples underwent filtration through a 0.45-µm nylon membrane prior to analysis.

### 3.5. Entrapment Efficiency

Col-CS-AuNPs were centrifuged at 10,000 rpm for 25 min to separate free colistin from Col-CS-AuNPs. The supernatant (free colistin) was collected to determine the colistin content. The percentage of colistin entrapped was determined using the indirect entrapment efficiency Equation (1):

(1) $$\mathrm{Entrapment}\;(\%)=\frac{\;\mathrm{amount}\mathrm{of}\;\mathrm{colistin}\;\mathrm{added}\;-\;\mathrm{amount}\;\mathrm{of}\;\mathrm{colistin}\;\mathrm{supernatant}}{\;\mathrm{amount}\;\mathrm{of}\;\mathrm{colistin}\;\mathrm{added}\;}\times 100$$

### 3.6. Drug Release from Col-CS-AuNPs

Cellulose membrane dialysis tubes with a cut-off diameter of 12,000 kDa (Thermo Fisher Scientific) were loaded with Col-CS-AuNPs (the total volume used in the synthesis method was approximately 67 mL) and immersed in beakers containing 500 mL of release medium (phosphate buffered saline pH 7.4 or 4.5).

The released medium was continuously stirred at room temperature (25–30 °C) using a magnetic stirrer. To maintain a constant volume, 3 mL of the released medium was withdrawn at each sampling interval and replaced with an equal volume of freshly released medium. The colistin concentration in each sample was quantified via HPLC analysis following the procedure described in the previous section. The percentage of cumulative drug release was determined using Equation (2) as follows:

(2) $$\mathrm{Cumulative}\;\mathrm{drug}\;\mathrm{release}\;(\%)=\frac{{\left[\mathrm{Drug}\right]}_{t}}{{[\mathrm{Drug}]}_{\mathrm{total}}}\times 100$$

[Drug]_t_ represents the concentration of released colistin at a specific time t, and [Drug]_total_ refers to the total colistin loaded onto the Col-CS-AuNPs.

### 3.7. Mechanism of Drug Release from Col-CS-AuNPs

Four kinetic models, including zero-order, first-order, simplified Higuchi, and Korsmeyer–Peppas, were used to determine the mechanism of the in vitro colistin release patterns. The zero-order kinetic model indicated that drug dissolution is unaffected by drug concentration, as shown in Equation (3):

Q_t_ = Q_0_ + *k*_0_t (3)

where Q_t_ represents the quantity of released drugs at a specific time t, Q_0_ represents the initial amount of drug in the solution, and *k_0_* refers to the rate constant of the zero-order reaction. A zero-order kinetic model was derived by plotting the cumulative percentage of the drug released against time [27].

The first-order model describes drug release occurring in a concentration-dependent manner Equation (4):

(4) $$\frac{\mathrm{dC}}{\mathrm{dt}}=-kC$$

where C represents the concentration of the drugs, and *k* represents the first-order rate constant. This differential equation can be expressed in a simplified form, as shown in Equation (5):

(5) $$\log\;C=\log\;C_{0}-\frac{-k}{2.303}$$

where C_0_ is the initial drug concentration. The first-order kinetic model was evaluated by plotting the log-cumulative percentage of the remaining drug against time [27].

The simplified Higuchi model uses the following equation to elucidate the process of drug release from the matrix and polymeric systems, as shown in Equation (6):

(6) $$\frac{M_{t}}{M_{\infty }}=k\sqrt{t}$$

where (M_t_/M_∝_) represents the total amount of drug released at time t, whereas *k* is the Higuchi constant specific to the formulation. Plotting the cumulative percentage of the drug released against the square root of time facilitated the evaluation of the Higuchi model [27].

The Korsmeyer–Peppas model describes the release of drugs from a matrix and polymeric system, as shown in Equation (7):

(7) $$\frac{M_{t}}{M_{\infty }}=K^{\prime }t^{n}$$

where (M_t_/M_∝_) is the total amount of drug released at time t, *K*′ is the kinetic constant, and the exponent n describes a particular diffusion mechanism. The Korsmeyer–Peppas model was evaluated by plotting the log-cumulative percentage of the drug released against the log of time [27].

Each model was plotted using Microsoft Excel (Microsoft Corp., Redmond, WA, USA), and a linear regression fit was performed to determine the rate constants and correlation values.

### 3.8. Preparation of Col-CS-AuNP MDI Formulation

The formulation process for the Col-CS-AuNP MDI, outlined in Table 5, began by accurately weighing Col-CS-AuNPs equivalent to 20 mg of colistin and dispersing them in 1 g of absolute ethanol to produce a concentrated suspension. Subsequently, sorbitan monooleate (0.005 g) was added as a surfactant to prevent drug aggregation and as a valve lubricant. The resulting product was then transferred to glass canisters (Schott AG, Mainz, Germany) with uncoated inner walls. Metering valves (50 µL; Bespak Europe, Ltd., Norfolk, UK) were used to seal the canisters, which were crimped using an aerosol crimping machine (model 2016, Pamasol Willi Mader, Zurich, Switzerland). After sealing and capping the valve, the HFA-134a propellant was filled into the canisters through the valve using an aerosol-filling machine (Pamasol Willi Maeder), yielding 10 mL of the Col-CS-AuNP MDI formulation. Finally, the completed MDIs were thoroughly mixed using a vortex before being stored at room temperature (25–35 °C) under light-protected conditions.

Each canister, MDI loaded with 10 mL of Col-CS-AuNP formulation, equivalent to 20 mg colistin, and equipped with a 50-μL metering valve, delivers a total of 200 puffs (100 µg of colistin per actuation). Table 6 presents the composition of the Col-CS-AuNP MDI.

### 3.9. Aerosol Properties of Col-CS-AuNP MDI Formulation

The aerosol performance of Col-CS-AuNP MDI was determined using the USP apparatus 1, BP apparatus D, or ACI without a pre-separator (Copley Scientific, Nottingham, UK) [76]. The equipment for MDI performance testing was assembled in accordance with USP General Chapter 601, as specified [76]. Eight ACI stages (stages 0–7) were connected to the induction port, and a mouthpiece adapter was used to achieve an airtight seal. A vacuum pump was installed in the vacuum tubing following the ACI filter stage, enabling control of the airflow at a flow rate of 28.3 L/min ± 5% through the system. After shaking the Col-CS-AuNP MDI for 5 s, the first two dosages were dispensed to waste. After that, the Col-CS-AuNP-loaded MDI was placed in the mouthpiece adapter while the vacuum pump was operated. The valve was actuated for 5–10 s to ensure complete dose discharge, meeting the minimal recommended dosage.

The MDI canister was detached from the mouthpiece adapter and vigorously shaken for 5 s. It was then re-inserted into the mouthpiece adapter and immediately activated by pressing the actuator. This process was repeated five times to ensure proper priming of the MDI. Additionally, to ensure an adequate drug concentration for analysis in the lower ACI stages, five doses of the Col-CS-AuNP MDI were discharged into the ACI. After discharging the last dose, the Col-CS-AuNP MDI canister was removed from the mouthpiece adapter. The induction port, mouthpiece adapter, and all stages of the ACI (stage 0 to filter) were washed and rinsed individually with the mobile phase to collect the drug deposited in each stage. Colistin deposited in each portion was quantified using HPLC following the procedure described in the previous section. The aerosol characteristics, including the FPF, FPD, MMAD, and GSD values, were obtained by generating a graph according to the procedures outlined in the pharmacopeia [76].

### 3.10. Antimicrobial Activity of Col-CS-AuNP MDI Product Concentrate

*E. coli* TISTR 887, obtained from the TISTR, was streaked on Muller–Hinton agar (MHA) and incubated for 24 h at 37 °C. The individual colonies obtained were inoculated into a tube containing 1 mL of CAMHB. An aliquot of 250 µL was transferred into 15 mL of CAMHB and mixed well. The turbidity of the bacterial suspension was adjusted to 0.1 at an OD of 625 nm (this 0.1 turbidity is equivalent to a standardized inoculum of 10^8^ CFU/mL). A dilution ratio of 1:20 was used to obtain 5 × 10^5^ CFU/mL. The MIC was determined using a broth microdilution assay. The colistin, Col-CS-AuNP, CS-AuNP product concentrate, and Col-CS-AuNP product concentrate (formulation without propellant) were prepared by conducting a two-fold serial dilution with CAMHB broth, covering an equivalent dose of colistin in the concentration range of 0–128 μg/mL. Each well was introduced with 100 µL of diluted samples and 10 µL of inoculum, and each concentration was studied in triplicate. As a microbiological quality control, CAMHB broth was employed; as a negative control, CAMHB broth containing the bacterial inoculum was used as a microbial growth control. CAMHB combined with colistin served as a positive control. Within the range of concentrations tested, the highest concentration of additives in the product was 0.6% *w*/*v* for ethanol, while the concentration of sorbitan monooleate was extremely low. These concentrations were insufficient to cause a significant antimicrobial effect. The 96-well plates were incubated at 37 °C for 16–24 h. The MIC was defined as the concentration at which no visible bacterial growth was observed on the microplate.

### 3.11. Time-Kill Kinetic Assay

The *E. coli* culture in CAMHB was standardized to an optical density of 0.1 at 625 nm. The cell suspension was diluted 1000 times before use. Col-CS-AuNP product concentrates at concentrations of 1, 5, and 10× MIC were prepared in a medium and further diluted 20-fold by transferring 50 μL aliquots to 1000 μL. The mixture was incubated for 0, 1, 2, 3, 6, 12, 18, or 24 h at 37 °C. The mixture incubated at each time point (10 μL) was 10-fold serially diluted with CAMHB. Additionally, MHA was plated with 10 μL of the serially diluted sample and incubated for 24 h at 37 °C. The number of bacterial colonies was quantified and expressed as CFU/mL [90]. The limit of detection of the plate count method was 100 CFU/mL (2 Log). Each test was performed in triplicate to ensure reliability. To identify significant differences (*p* < 0.05) between the treated and untreated samples, a one-way analysis of variance (ANOVA) was performed.

### 3.12. Cell Viability Test

#### 3.12.1. Cell Culture Conditions

The A549 cell line (ATCC: CCL185), derived from human lung adenocarcinoma, was cultured in Kaighn’s modification of Ham’s F-12K (Kaighn’s) Medium supplemented with 10% fetal bovine serum (FBS), 100 U penicillin, and 100 U/mL streptomycin. The cells were incubated at 37 °C with 5% CO_2_. The medium was changed every second day. Human Caucasian bronchioalveolar carcinoma cells (NCI-H358; ECACC, Wiltshire, UK) were cultured in Roswell Park Memorial Institute (RPMI) 1640 medium containing 2 mM glutamine, 10% FBS, 100 U penicillin, and 100 U/mL streptomycin under 5% CO_2_ at 37 °C. The medium was changed every other day.

#### 3.12.2. Cytotoxicity Test

Human Caucasian bronchioalveolar carcinoma (NCI-H358) and human lung adenocarcinoma (A549) cells were introduced at a cell density of 1 × 10^5^ cells/mL, 100 μL per well in a 96-well plate. Following a 24-h incubation period, colistin, Col-CS-AuNP product concentrate, and CS-AuNP product concentrate equivalent to a colistin concentration in the range of 3.12–200 µg/mL in fresh media were added to each well of a 96-well plate and incubated for 24 h. CS-AuNP-treated cells were used as the negative control. The concentration of CS-AuNPs was calculated from the colistin equivalent weight of the Col-CS-AuNPs. Standard colistin was used as a positive control. Within the range of concentrations tested, the highest concentration of additives in the product was 1% *w*/*v* for ethanol, while the concentration of sorbitan monooleate was extremely low. These concentrations were insufficient to cause cytotoxicity. Following a 24 h exposure, cell viability was assessed using MTT. Briefly, cells were treated with 80 µL of fresh medium and 20 µL of MTT solution and incubated at 37 °C with 5% CO_2_ for 4 h. Following the complete incubation period, the MTT-containing medium was discarded, and dimethyl sulfoxide (100 µL) was added to dissolve the formed formazan salt. The absorbance was measured at 570 nm using a microplate reader (Biohit 830, Biohit^®^, Helsinki, Finland). The percentage of cell viability was calculated compared to the negative untreated control using the following equation Equation (8).

(8) $$\%\;\mathrm{cell}\;\mathrm{viability}=\frac{Abs\;570\;nm\;of\;sample\;treated\;cell}{Abs\;570\;nm\;of\;untreated\;cell}\times 100$$

### 3.13. Stability Study

Colloidal Col-CS-AuNPs were stored in polyethylene tubes at 2–8 °C for 3 months. At the end of the storage period, the appearance, colistin content, size, and zeta potential of the Col-CS-AuNPs were recorded. A student’s *t*-test (*p* < 0.05) was used to examine the differences in drug content, particle size, and zeta potential following storage. Each experiment was performed in triplicate. Col-CS-AuNP MDI formulations were stored under long-term conditions at 30 ± 0.5 °C and relative humidity (RH) of 75% ± 5%, in accordance with the ASEAN Guidelines on Stability Study of Drug Products [89]. Due to study period constraints, the MDI products were examined and recorded for 3 months; however, their long-term stability remains under investigation. The assay content and uniformity of colistin content in the Col-CS-AuNP formulation for MDI were assessed. Additionally, the aerosol properties of the formulation, including MMAD, GSD, ED, FPD, and FPF, were also evaluated at the end of the storage period.

## 4. Conclusions

Col-CS-AuNPs were successfully synthesized with an average of 76.4% drug loading efficacy. The observed physicochemical properties of the Col-CS-AuNPs rendered them acceptable as nanoparticles. The colistin content, particle size, and zeta potential of the Col-CS-AuNPs remained unchanged when stored at 2–8 °C. Colistin was released from Col-CS-AuNPs in a prolonged manner, reaching 85% drug concentration. The release of colistin was best described by the Korsmeyer–Peppas model owing to the polymer system. When developed as an MDI formulation, Col-CS-AuNPs exhibited suitable aerosol properties for pulmonary drug delivery. Moreover, they remained stable throughout the 3-month study period at 30 ± 2 °C and 75% ± 5% RH. Further, the formulated MDI effectively killed bacteria over a 12-h period at 10× MIC. Long-term sustained release of the drug from the developed MDI formulation suggested its suitability for therapeutic use against bacterial respiratory infections. However, the significantly lower toxicity of the Col-CS-AuNP MDI formulation than colistin, as observed in both upper and lower respiratory tract cell lines, indicates its potential for treating lung infections. Furthermore, additional animal studies are required to collect adequate data regarding the efficacy and safety of MDI loaded with Col-CS-AuNP formulations.

## Figures and Tables

**Table 1:** Physical properties of gold nanoparticles (AuNPs), chitosan-capped gold nanoparticles (CS-AuNPs), and colistin conjugated with chitosan-capped gold nanoparticles (Col-CS-AuNPs). Data are presented as mean ± SD, *n* = 4.

Particles	Particle Size, Z-Average (nm)	Polydispersity Index (PDI)	Zeta Potential (mV)
CS-AuNPs	44.34 ± 1.02	0.22 ± 0.01	+1.13 ± 0.01
Col-CS-AuNPs	174.50 ± 4.46	0.10 ± 0.01	+4.97 ± 1.05

**Table 2:** Rate constants and correlation coefficients were obtained by modeling colistin release from Col-CS-AuNPs using the following: zero-order kinetic model, first-order kinetic model, simplified Higuchi model, and Korsmeyer–Peppas model.

Model of Drug Release	Rate Constant		Correlation Coefficients (R^2^)
Zero-order	*k_0_*	0.1794	0.4951
First-order	*k_1_*	0.0024	0.7563
Higuchi	*k_H_*	3.2782	0.6646
Korsmeyer–Peppas	*n*	0.1201	0.9531

**Table 3:** The aerosol properties of Col-CS-AuNP MDI formulations (mean ± SD, *n* = 6).

Test Parameters	Results
Assay (% Labeled claim of colistin)	98.64% ± 2.34
Delivered dose uniformity	BOU: 95.05% ± 4.08% EOU: 96.45% ± 1.33%
MMAD (μm)	2.34 ± 1.01
GSD	0.21 ± 0.02
ED (μg)	88.5 ± 1.85
FPF (%)	61.08 ± 2.03
FPD (μg)	61.08 ± 2.03

Abbreviations: Beginning of unit life (BOU), end of unit life (EOU), mass median aerodynamic diameter (MMAD), emitted dose (ED), fine particle fraction (FPF), fine particle dose (FPD), and geometric standard deviation (GSD).

**Table 4:** MIC and MBC of colistin and Col-CS-AuNPs (mean ± SD, *n* = 3).

Sample	MIC (μg/mL) against *E. coli* TISTR 887	MBC (μg/mL) against *E. coli* TISTR 887
Colistin	4 ± 0.00	4 ± 0.00
Col-CS-AuNPs	8 ± 0.00	8 ± 0.00
CS-AuNPs	128 ± 0.00	128 ± 0.00
Col-CS-AuNP MDI product concentrate	8 ± 0.00	8 ± 0.00

**Table 5:** The stability results of Col-CS-AuNPs after storage at 2–8 °C and Col-CS-AuNP MDI formulation after storage at 30 ± 2 °C and relative humidity of 75% ± 5% for 3 months. The data are shown as the mean ± SD, *n* = 3–6.

Test Parameters	Initial Results	After 3 Months of Storage
Col-CS-AuNPs (stored at 2–8 °C)		
Appearance	Red wine color colloidal solution	Red wine color colloidal solution
Content of colistin (%*w*/*w*)	2.84 ± 0.50	2.79 ± 0.21
Particle size (Z-average, nm)	174.50 ± 4.46	186.30 ± 3.65
Polydispersity index	0.10 ± 0.01	0.12 ± 0.02
Zeta potential (mV)	4.97 ± 1.05	5.74 ± 0.85
Col-CS-AuNP MDI formulation (stored at 30 ± 2 °C/75 ± 5% relative humidity)		
Assay (%Labeled claim of colistin)	98.64 ± 2.34	101.52 ± 1.40
Delivered dose uniformity	BOU: 95.05% ± 4.08% EOU: 96.45% ± 1.33%	BOU: 92.34% ± 1.70% EOU: 94.10% ± 2.09%
MMAD (μm)	2.34 ± 1.01	2.87 ± 1.74
GSD	0.21 ± 0.02	0.22 ± 0.01
ED (μg)	17.70 ± 1.22	20.14 ± 1.68
FPF (%)	61.08 ± 2.03	55.48 ± 3.10
FPD (μg)	61.08 ± 2.03	55.48 ± 3.10

Abbreviations: Beginning of unit life (BOU), end of unit life (EOU), mass median aerodynamic diameter (MMAD), emitted dose (ED), fine particle fraction (FPF), fine particle dose (FPD), and geometric standard deviation (GSD).

**Table 6:** Composition of the Col-CS-AuNPs suspension MDI formulation per canister (total of 200 doses).

Ingredients	Amount (g)	Function
Col-CS-AuNPs (equivalent to colistin 0.02 g) *	0.700	Active ingredient
Absolute ethanol	1.00	Vehicle of product concentrate
Sorbitan monooleate	0.005	Surfactant (valve lubricant and stabilizing agent)
HFA-134a qs to	10.00 mL	Propellant

* Note: the 0.7 g of Col-CS-AuNPs contained 0.02 g of pure colistin, as determined by the entrapment efficiency calculation. The Col-CS-AuNP MDI formulation was equivalent to 200 doses; therefore, 1 puff (50 μL) contained 100 μg of colistin.

## Data Availability

All data generated or analyzed during this study are included in this published article.

## Supplementary Materials

The following supporting information can be downloaded at: https://www.mdpi.com/article/10.3390/antibiotics13070630/s1, Figure S1: The appearance of gold (III) chloride (HAuCl4) solution (A), chitosan-capped gold nanoparticles (CS-AuNPs) colloidal solution (B), and colistin conjugated with chitosan-capped gold nanoparticles (Col-CS-AuNPs) colloidal solution during synthesis (C); Figure S2: UV-Vis spectra between 200 and 700 nm of gold (III) chloride (HAuCl_4_) solution (blue line), chitosan-capped gold nanoparticles (CS-AuNPs) colloidal solution (green line), and colistin conjugated with chitosan-capped gold nanoparticles (Col-CS-AuNPs) colloidal solution (red line); Figure S3: Dynamic light scattering (DLS) particle size distribution of chitosan-capped gold nanoparticles (CS-AuNPs) colloidal solution (A), and colistin conjugated with chitosan-capped gold nanoparticles (Col-CS-AuNPs) (B); Figure S4: The FT-IR spectra of colistin sulfate, chitosan-capped gold nanoparticles (CS-AuNPs), and colistin conjugated with chitosan-capped gold nanoparticles (Col-CS-AuNPs); Figure S5: 500 MHz ^1^H NMR spectrum of colistin (A), Col-CS-AuNPs (chemical shift 0–3; B) and Col-CS-AuNPs (chemical shift 3–6; C) in D_2_O; Figure S6: HPLC chromatogram of standard colistin sulfate at a concentration of 50 μg/mL (red line) and colistin sulfate collected from supernatant of the synthesis Col-CS-AuNPs (black line) with UV detector at a wavelength of 215 nm. The retention time of standard and sample colistin were eluted at the same time of 9.5 min; Table S1: Summary of ^1^H NMR resonance of colistin and Col-CS-AuNPs; Table S2: Elemental analysis of colistin and AuNPs (mean ± SD, *n* = 3).

## Abbreviations

ACI: Andersen Cascade Impactor; API, Active pharmaceutical ingredient; ASEAN, The Association of Southeast Asian Nations; ATR, attenuated total reflection; AuNPs, gold nanoparticles; BOU, beginning of unit life; CAMHB, cation-adjusted Mueller–Hinton broth; CFU, colony-forming unit; Col-CS-AuNPs, colistin-chitosan conjugated on gold nanoparticles; CS-AuNPs, chitosan conjugated on gold nanoparticles; DLS, dynamic light scattering; DPI, dry powder inhaler; ED, emitted dose; EOU, end of unit life; FBS, fetal bovine serum; FPD, fine particle dose; FPF, fine particle fraction; FT-IR, Fourier-transform infrared spectroscopy; GSD, geometric standard deviation; HFA134a, 1,1,1,2-tetrafluoroethane; HPLC, high-performance liquid chromatography; LOD, limit of detection; LOQ, limit of quantitation; LPS, lipopolysaccharide; LSPR, localized surface plasmon resonance; MCR, mobilizable colistin resistance; MDI, metered-dose inhaler; MDR, multi-drug resistant; MH, Muller–Hinton; MIC, minimum inhibitory concentration; MMAD, mass median aerodynamic diameter; MTT, 3-(4, 5-dimethylthiazol-2-yl)-2,5-diphenyl-tetrazolium bromide; NMR, nuclear magnetic resonance; PDI, poly-dispersity index; rpm, round per minute; SEM, scanning electron microscope; TISTR, Thailand Institute of Scientific and Technological Research; USP, The United States Pharmacopoeia; UV, ultraviolet; UV-Vis, ultraviolet-visible.
