# Supplementary material for: In Vitro Evaluation of Colistin Conjugated with Chitosan-Capped Gold Nanoparticles as a Possible Formulation Applied in a Metered-Dose Inhaler
Source: Antibiotics (Basel). 2024 Jul 6;13(7):630. doi: 10.3390/antibiotics13070630 (PMC11274357; doi:10.3390/antibiotics13070630)
Supplement: Supplementary file 1 [file antibiotics-13-00630-s001.zip › antibiotics-3015487-supplementary.pdf]

Supporting Information

In vitro Evaluation of Colistin Conjugated with Chitosan-Capped Gold Nanoparticles as Possible Formulation Applied in Metered-Dose Inhaler

Narumon Changsan [1], Apichart Atipairin [2,3], Poowadon Muenraya [2,3], Rutthapol Sritharadol [4], Teerapol Srichana [5], Neelam Balekar [6], Somchai Sawatdee [2,3,*]

1. College of Pharmacy, Rangsit University, Pathumtani 12000, Thailand; narumon.c@rsu.ac.th (N.C.)
2. School of Pharmacy, Walailak University, Thasala, Nakhon Si Thammarat 80160, Thailand; apichart.at@mail.wu.ac.th (A.A.); poowadon.me@wu.ac.th (P.M.)
3. Drug and Cosmetics Excellence Center, Walailak University, Thasala, Nakhon Si Thammarat 80160, Thai-land
4. Department of Pharmaceutics and Industrial Pharmacy, Faculty of Pharmaceutical Sciences, Chulalongkorn University, Bangkok 10330, Thailand; rutthapol.s@chula.ac.th (R.S.)
5. Drug Delivery System Excellence Center, Department of Pharmaceutical Technology, Faculty of Pharma-ceutical Sciences, Prince of Songkla University, Hat Yai, Songkhla 90112, Thailand; teerapol.s@psu.ac.th (T.S.)
6. College of Pharmacy, IPS Academy, Indore, Madhya Pradesh 452012, India; neelambalekar@gmail.com (N.B.)

*Correspondence: somchai086@hotmail.com or somchai.sa@wu.ac.th (S.S.)
Tel.: +66 (0) 75672818; Fax: +66 (0) 75672814

Supplement Figures

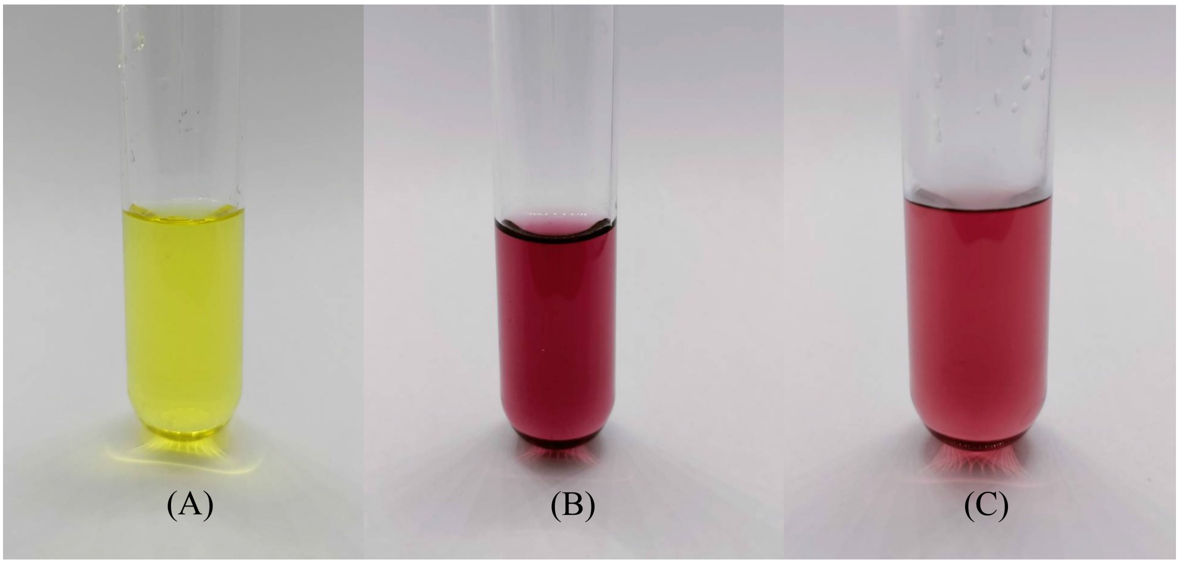

Figure S1. The appearance of gold (III) chloride (HAuCl4) solution (A), chitosan-capped gold nanoparticles (CS-AuNPs) colloidal solution (B), and colistin conjugated with chitosan-capped gold nanoparticles (Col-CS-AuNPs) colloidal solution during synthesis (C).

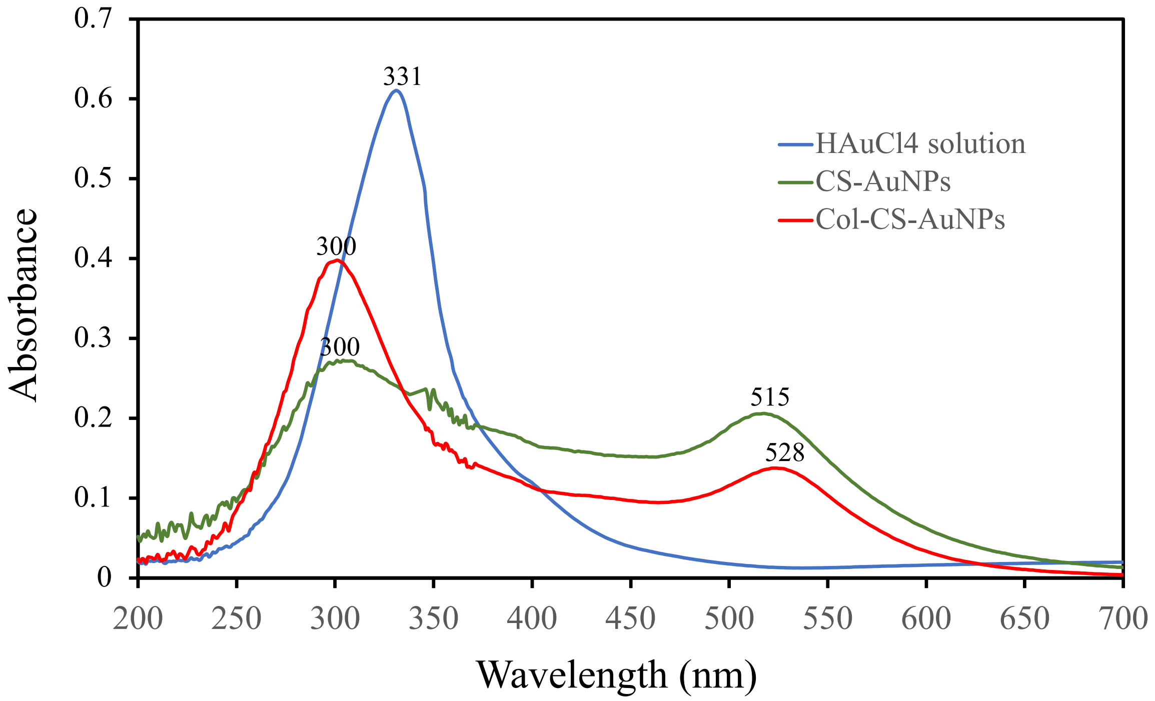

Figure S2. UV-Vis spectra between 200 and 700 nm of gold (III) chloride ($HAuCl_4$) solution (blue line), chitosan-capped gold nanoparticles (CS-AuNPs) colloidal solution (green line), and colistin conjugated with chitosan-capped gold nanoparticles (Col-CS-AuNPs) colloidal solution (red line).

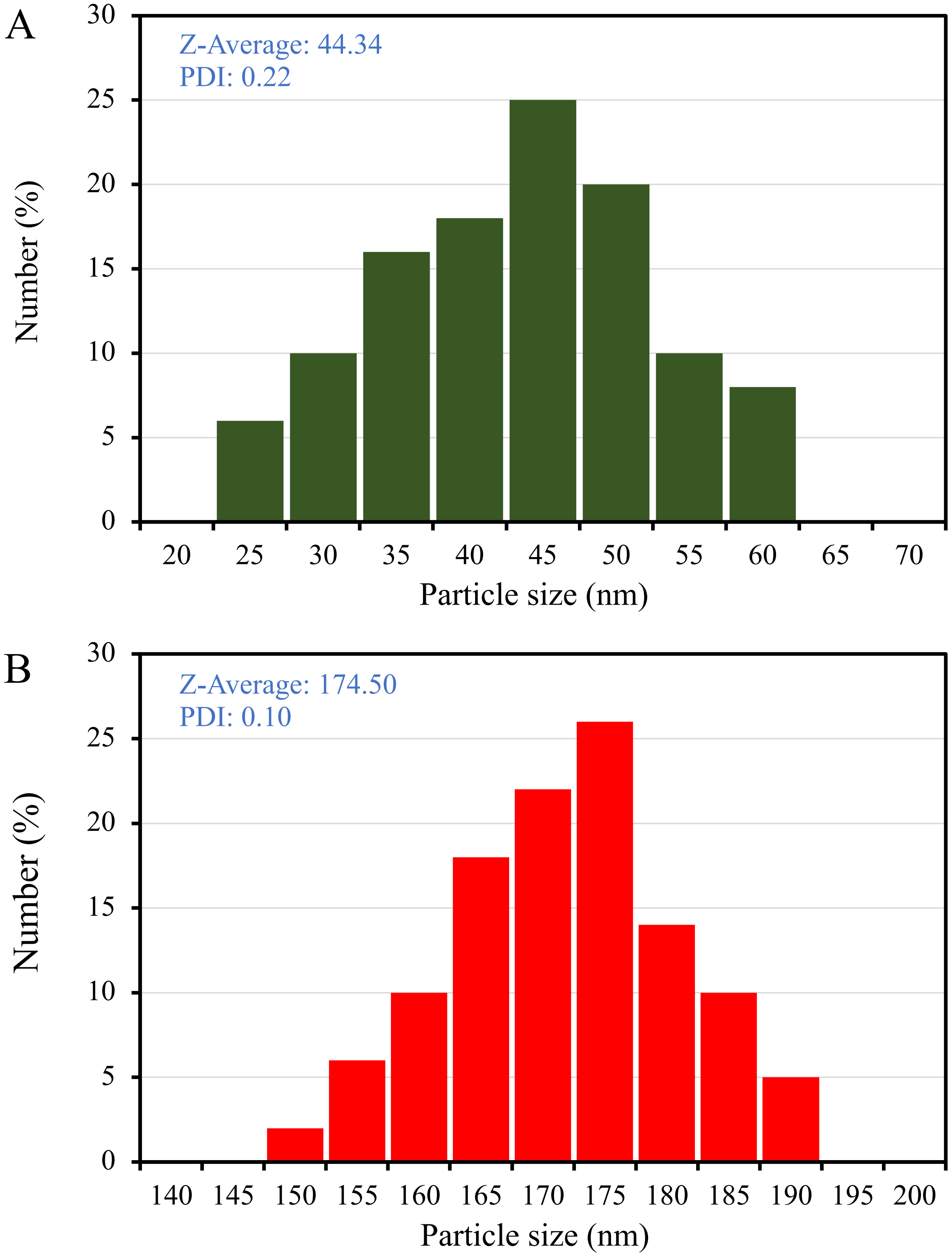

Figure S3. Dynamic light scattering (DLS) particle size distribution of chitosan-capped gold nanoparticles (CS-AuNPs) colloidal solution (A), and colistin conjugated with chitosan-capped gold nanoparticles (Col-CS-AuNPs) (B).

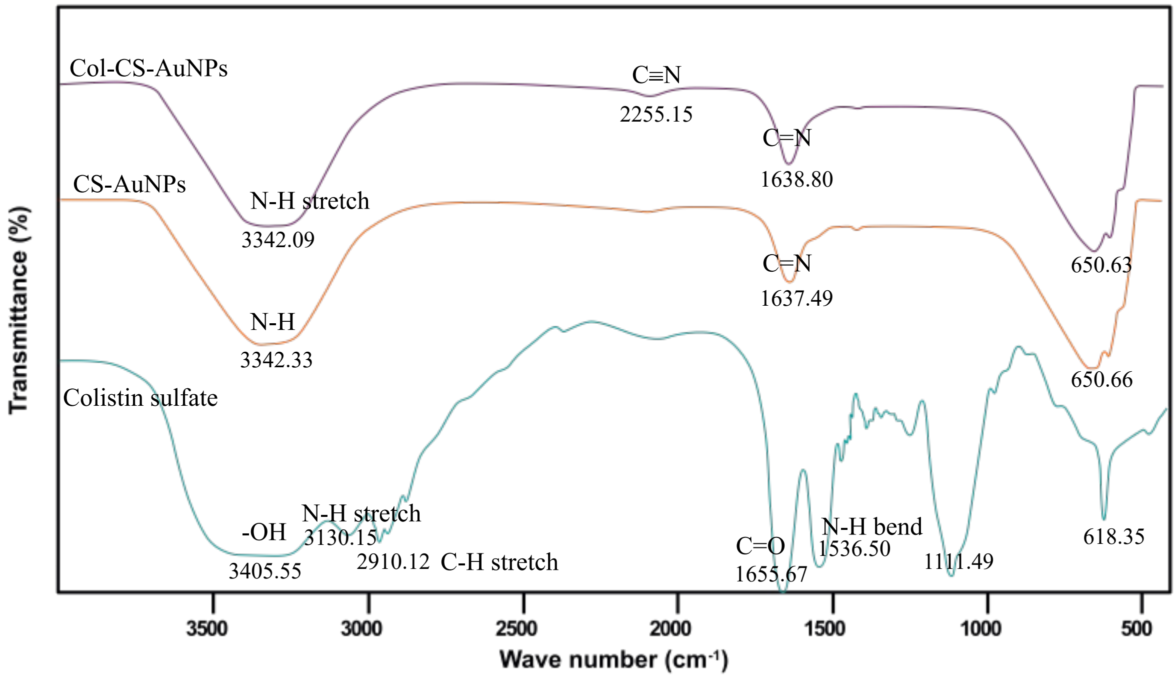

Figure S4. The FT-IR spectra of colistin sulfate, chitosan-capped gold nanoparticles (CS-AuNPs), and colistin conjugated with chitosan-capped gold nanoparticles (Col-CS-AuNPs).

A.

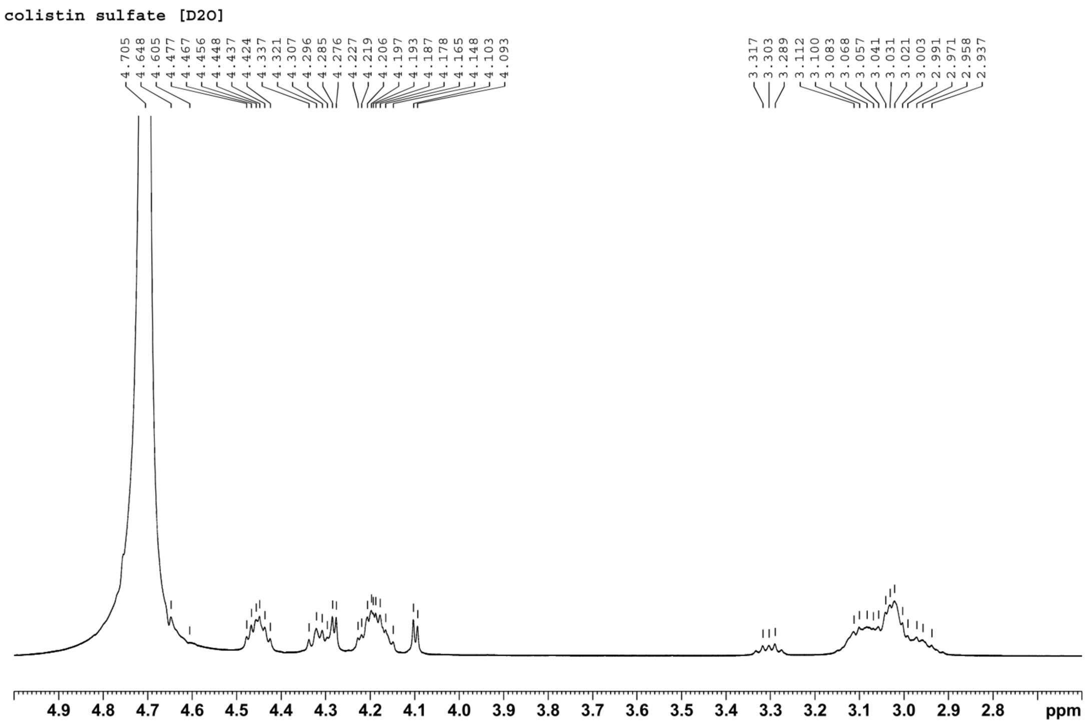
colistin sulfate [D2O]
4.705
4.648
4.605
4.477
4.467
4.456
4.448
4.437
4.424
4.337
4.321
4.307
4.296
4.285
4.276
4.227
4.219
4.206
4.197
4.193
4.187
4.178
4.165
4.148
4.103
4.093
3.317
3.303
3.289
3.112
3.100
3.083
3.068
3.057
3.041
3.031
3.021
3.003
2.991
2.971
2.958
2.937
4.9 4.8 4.7 4.6 4.5 4.4 4.3 4.2 4.1 4.0 3.9 3.8 3.7 3.6 3.5 3.4 3.3 3.2 3.1 3.0 2.9 2.8 ppm

B.

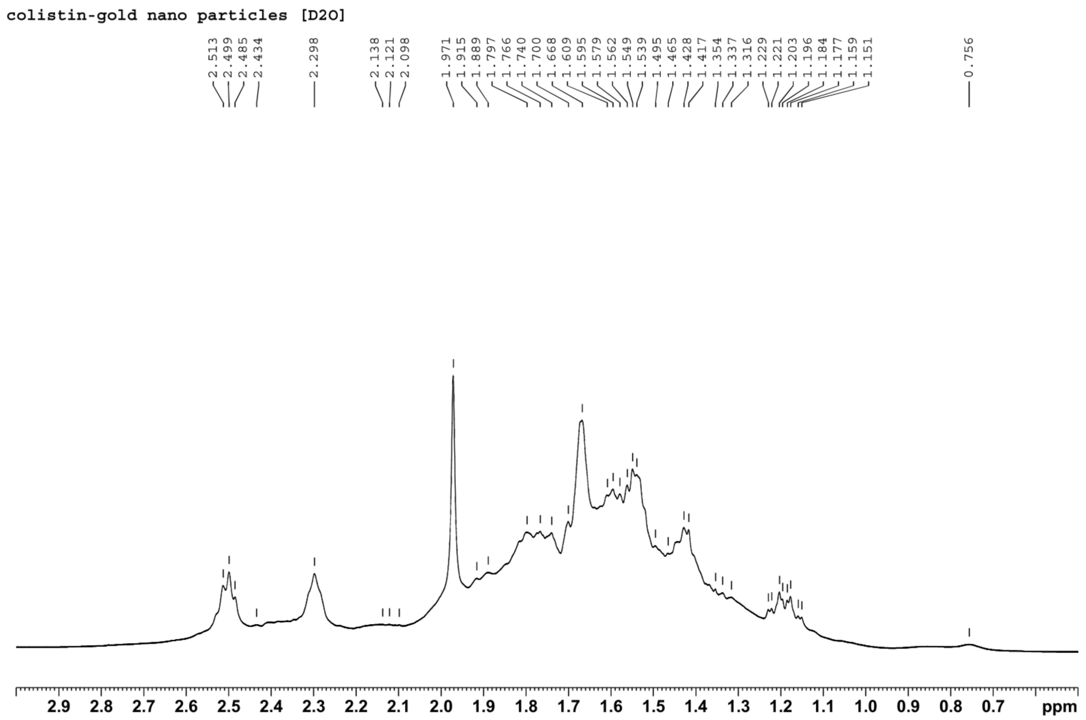
colistin-gold nano particles [D2O]
2.513
2.499
2.485
2.434
2.298
2.138
2.121
2.098
1.971
1.915
1.889
1.797
1.766
1.740
1.700
1.668
1.609
1.595
1.579
1.562
1.549
1.539
1.495
1.465
1.428
1.417
1.354
1.337
1.316
1.229
1.221
1.203
1.196
1.184
1.177
1.159
1.151
0.756
2.9 2.8 2.7 2.6 2.5 2.4 2.3 2.2 2.1 2.0 1.9 1.8 1.7 1.6 1.5 1.4 1.3 1.2 1.1 1.0 0.9 0.8 0.7 ppm

C.

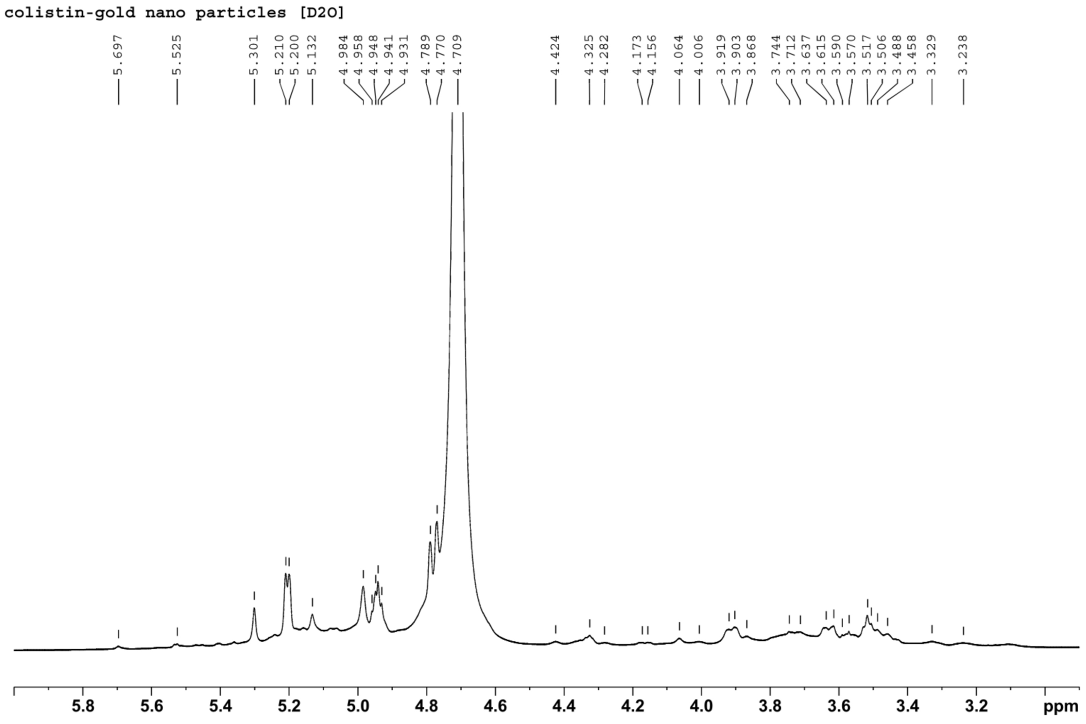

Figure S5. 500 MHz ^{1}H NMR spectrum of colistin (A), Col-CS-AuNPs (chemical shift 0-3; B) and Col-CS-AuNPs (chemical shift 3-6; C) in D_2O.

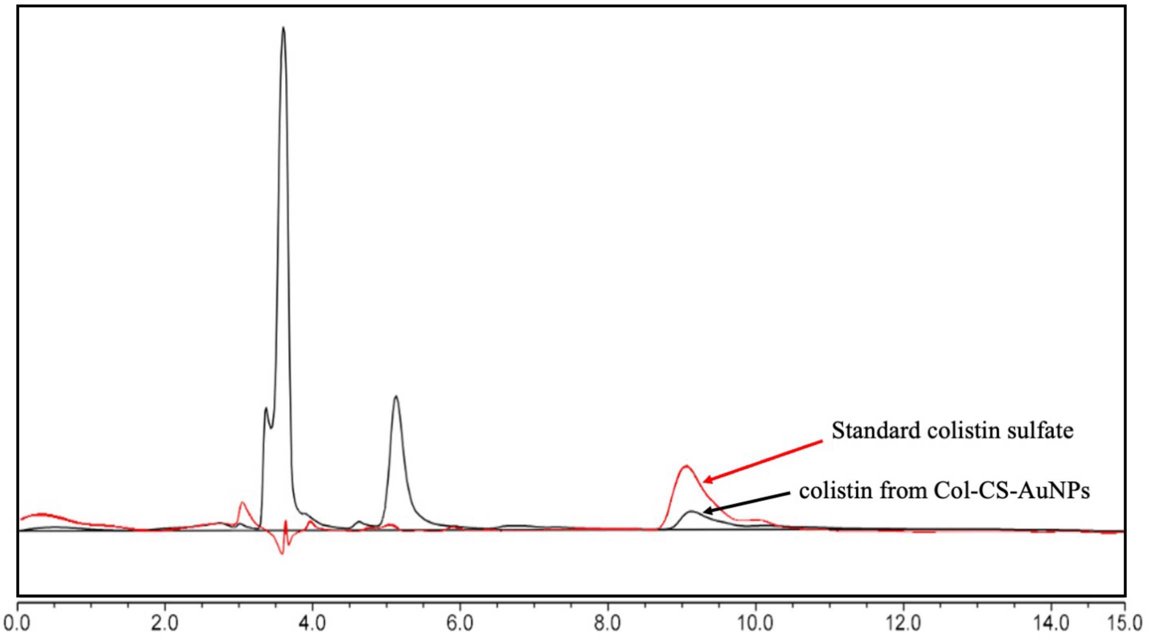

Figure S6. HPLC chromatogram of standard colistin sulfate at a concentration of 50 µg/mL (red line) and colistin sulfate collected from supernatant of the synthesis Col-CS-AuNPs (black line) with UV detector at a wavelength of 215 nm. The retention time of standard and sample colistin were eluted at the same time of 9.5 min.

Supplement Tables

Table S1. Summary of ^{1}H NMR resonance of colistin and Col-CS-AuNPs.

Group	Chemical shift (ppm)	
	Colistin sulfate	Col-CS-AuNPs
Methyl from fatty acid	0.81	0.76
Methyl from secondary alcohol	1.16	1.19
Alcohol	1.47	1.43
Amine primary	1.60	1.56
Amine primary	-	1.78 *
Methylene	2.04	1.97
Methylene	2.17	2.31
Methylene from amine primary	2.25	2.51
Methine	3.04	3.51
Alcohol	-	3.70 *
Methylene from secondary amide	4.18	4.78
Methine from secondary amide	4.30	4.95
Secondary amide	7.73	6.68

The asterisk (*) indicates broad peak with multigroup.

Table S2. Elemental analysis of colistin and AuNPs (mean ± SD, n = 3).

**Sample**	**Percentage of elemental**			
	**C (%)**	**H (%)**	**N (%)**	**O (%)**
Colistin	52.1 ± 0.04	8.1 ± 0.19	18.1 ± 0.03	21.7 ± 0.42
CS-AuNPs	44.1 ± 0.06	6.8 ± 2,12	8.1 ± 0.03	41.0 ± 0.44
Col-CS-AuNPs	45.9 ± 0.04	7.6 ± 1.46	10.2 ± 0.05	36.3 ± 0.44
